# Retinoid X receptor agonist 9CDHRA mitigates retinal ganglion cell apoptosis and neuroinflammation in a mouse model of glaucoma

**DOI:** 10.1096/fj.202402642R

**Published:** 2025-03-13

**Authors:** Devaraj Basavarajappa, Nitin Chitranshi, Seyed Shahab Oddin Mirshahvaladi, Veer B. Gupta, Viswanthram Palanivel, Gabriella E. Parrilla, Akanksha Salkar, Mehdi Mirzaei, András M. Komáromy, Wojciech Krezel, Stuart L. Graham, Vivek Gupta

**Affiliations:** ^1^ Macquarie Medical School, Faculty of Medicine, Health and Human Sciences Macquarie University Sydney New South Wales Australia; ^2^ School of Medicine Deakin University Geelong Victoria Australia; ^3^ Department of Small Animal Clinical Sciences, College of Veterinary Medicine Michigan State University East Lansing Michigan USA; ^4^ Institut de Génétique et de Biologie Moléculaire et Cellulaire, Institut de la Santé et de la Recherche Médicale (U1258), Centre National de la Recherche Scientifique (UMR7104) Université de Strasbourg, Fédération de Médecine Translationnelle de Strasbourg Illkirch France

**Keywords:** 9CDHRA, apoptosis, ER stress, glaucoma, glial cell, intraocular pressure, neuroinflammation, neuroprotection, retinoid X receptors

## Abstract

Glaucoma, a leading cause of irreversible blindness, is characterized by the progressive loss of retinal ganglion cells (RGCs) and optic nerve damage, often associated with elevated intraocular pressure (IOP). Retinoid X receptors (RXRs) are ligand‐activated transcription factors crucial for neuroprotection, as they regulate gene expression to promote neuronal survival via several biochemical networks and reduce neuroinflammation. This study investigated the therapeutic potential of 9‐cis‐13,14‐dihydroretinoic acid (9CDHRA), an endogenous retinoid RXR agonist, in mitigating RGC degeneration in a high‐IOP‐induced experimental model of glaucoma. We administered 9CDHRA to glaucomatous mice eyes via intravitreal injections and assessed its effects on endoplasmic reticulum (ER) stress markers, glial cell activation, and RGC survival. Our findings demonstrated that 9CDHRA treatment significantly protected inner retinal function and retinal laminar structure in high‐IOP glaucoma. The treatment reduced ER stress markers, increased protein lysine acetylation, and diminished glial cell activation, leading to a significant decrease in apoptotic cells under glaucomatous conditions. These results suggest that 9CDHRA exerts neuroprotective effects by modulating key pathogenic pathways in glaucoma, highlighting its potential as a novel therapeutic strategy for preserving vision in glaucoma.

## Graphical abstract

In this study, we investigated the effects of the retinoid X receptor (RXR) natural ligand 9CDHRA on retinal structure and function using a mouse model of glaucoma induced by elevated intraocular pressure (IOP). Our findings demonstrate that 9CDHRA preserves retinal structure and function by suppressing the ER stress pathway, inhibiting apoptosis, and reducing neuroinflammatory activation of microglia and macroglia under glaucomatous conditions.

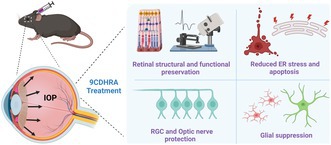

## 1 INTRODUCTION

Retinoid X receptors (RXRs) belong to a specific subgroup in the nuclear receptors (NRs) superfamily that profoundly influence gene expression and cellular functions. The RXR family consists of α, β, and γ isotypes, and they exhibit a unique ability to form homodimer or heterodimer complexes with other NRs, including retinoic acid receptors (RARs), lipid X receptor (LXR), peroxisome proliferator‐activated receptor (PPAR) and nuclear receptor‐related‐1 (Nurr1), influencing intricate signaling pathways.^1^, ^2^, ^3^ RXRs function as ligand‐activated transcription factors to regulate target gene expression and possess a multidomain molecular architecture with corresponding activities, including the N‐terminal activation function (AF‐1), the central core DNA‐binding domain (DBD), and the C‐terminal ligand‐binding domain (LBD).^4^ The conformational changes induced by a ligand binding determine the composition of the receptor dimer complex bound to specific DNA sequences known as retinoid response elements (RAREs) and in particular recruitment of coactivators to promote gene transcription.^4^, ^5^ Activation of RXRs can occur either through the binding of endogenous or exogenous ligands (rexinoids). Various derivatives of retinoids (e.g. 9‐cis retinoic acid (9‐cis‐RA)) and free fatty acids (e.g. docosahexaenoic acid (DHA) or eicosapentaenoic acid (EPA)) were suggested to function as endogenous ligands for RXRs in specific conditions.^6^ Among all the proposed endogenous ligands, 9‐cis‐RA has been considered an endogenous ligand of RXRs for a long time.^7^, ^8^ RARs, which are well‐studied and physiologically relevant partners of RXRs, are also activated by all‐trans retinoic acid or 9‐cis‐RA, further underscoring the broader regulatory biochemical networks governed by this interaction.^3^, ^9^ In 2015, 9‐cis‐13,14‐dihydroretinoic acid (9CDHRA) was reported as the first endogenous, physiologically relevant ligand of RXRs, and it represents an active metabolite of a novel vitamin A5/X pathway identified in rodents and humans.^10^, ^11^, ^12^, ^13^ Further, high physiological concentrations of 9CDHRA for transactivation of RXRs are believed to be achieved upon the administration of high doses of synthetic dihydro‐retinoids or after the intake of high amounts of foods rich in vitamin A5/X derivatives.^14^

Acting as a crucial transcriptional regulator, RXRs via heterodimer complex formation play multifaceted roles in the CNS, including the retina. They influence neurodevelopment, neurodifferentiation, neurotransmission, remyelination, neuroprotection, and immune modulation.^2^, ^15^, ^16^ In recent years, an increasing number of pieces of evidence show the implication of RXRs in various neurological disorders, including Alzheimer's disease, Parkinson's disease, multiple sclerosis, and stroke.^2^, ^15^, ^16^, ^17^ As a result, RXRs have become a promising focus for therapeutic interventions, and there has been a growing interest in the utilization of retinoids in preclinical models for developing treatments for neurodegenerative diseases.^18^ Activation of RXRs by specific ligands modulates targeted gene expression and their biochemical networks. These specific modulations have been shown to reduce endoplasmic reticulum (ER) stress and suppress proapoptotic pathways in neuronal stress and toxicity conditions.^19^ Beneficial effects of RXR activation have also been observed during mitochondrial stress and free radical accumulation. Further, ligand‐mediated modulation of RXRs has been shown to attenuate inflammation via STAT3, MAPK, and NF‐κB signaling pathways and enhance oligodendrocyte progenitor cell (OPC) differentiation to promote remyelination.^2^, ^18^, ^20^

Glaucoma is a multifaceted eye disease characterized by optic nerve damage and progressive degenerative loss of retinal ganglion cells (RGCs) within the inner retina, leading to irreversible vision loss. An important risk factor in glaucoma pathogenesis and disease progression is high intraocular pressure (IOP) mediated by biomechanical stress.^21^ However, patients with normal IOP also develop glaucoma, and in many high‐IOP patients, the vision continues to deteriorate even after IOP‐lowering management.^22^, ^23^ The underlying molecular mechanisms of glaucoma pathophysiology are presently not well understood. Dysregulated biochemical networks of pro‐survival and pro‐death signaling pathways are thought to strongly influence the degeneration of RGCs and induce optic nerve damage in glaucoma.^24^ RGC degeneration in glaucoma is mediated by multifaceted processes, including oxidative stress, mitochondrial dysfunction, neurotrophic factor deficits, excitotoxicity, ER stress, vascular dysfunction, and glial activation.^24^, ^25^, ^26^ Several studies have demonstrated the expression of retinoid RXRs in retinal cells and highlighted their crucial role in retinal development, as well as their dysregulation in retinal degenerative diseases, including glaucoma.^2^, ^24^, ^27^, ^28^ Our previous research has shown RXR expression in RGCs in both human and mouse retinas and documented its dysregulation in glaucoma.^19^ Furthermore, pharmacological activation of RXRs, such as with the agonist bexarotene, has been shown to prevent apoptosis in retinal injury models and cellular stress models by modulating ER stress and proapoptotic signaling pathways.^19^, ^29^ Developing effective neuroprotective treatments for glaucoma requires identifying key molecular targets within signaling pathways to prevent or delay RGC loss. In this regard, RXR modulation with potent ligands offers a promising approach to mitigate dysfunctional molecular pathways linked to RGC degeneration. RXR activation by specific ligands has been shown to regulate targeted gene expression and influence the biochemical networks to alleviate ER and mitochondrial stress and suppress proapoptotic and inflammatory pathways in neuronal injury models.^2^ Based on these findings, this study hypothesized that targeting RXR with its natural ligand could protect RGCs from degeneration in a glaucoma animal model. In this study, we identified the protective effects of 9CDHRA treatment in vivo, demonstrating its ability to mitigate RGC loss and optic nerve damage under elevated IOP conditions. The activation of RXRs by its natural ligand, 9CDHRA, induced a biochemical network that suppressed the ER stress pathway, inhibited apoptotic changes, restored key proteins involved in lipid homeostasis and membrane integrity, and suppressed the inflammatory activation of microglia and macroglia.

## 2 MATERIALS AND METHODS

### 2.1 Experimental animals and 9CDHRA treatment

All procedures involving animals in this study adhered to the Australian Code of Practice for the Care and Use of Animals for Scientific Purposes, as well as the guidelines outlined in the ARVO (Association for Research in Vision and Ophthalmology) Statement for the Use of Animals in Ophthalmic and Vision Research, and Macquarie University Animal Ethics Committee approved all experimental protocols (AEC Reference No. 2020/029). Four to 6‐week‐old wild‐type C57BL/6 mice were obtained from Animal Resources Centre (ARC), Perth, Australia, and Ozgene ARC Pty Ltd., Bentley, Western Australia, and kept in the Macquarie University Animal Care Facility under regulated temperature (between 21 and 28°C) and 12‐hour light/dark cycles. Three to four mice were housed in each cage. 9CDHRA was obtained from Acanthus Research Inc. (Mississauga, Canada; ACA‐160929‐0001, Lot# RETR‐9887‐S906‐108A, Purity 96.7%). The sterile solution of 9CDHRA was prepared as described previously.^10^ Briefly, 9CDHRA was dissolved in ethanol and sterile dimethylsulfoxide (DMSO) and then resuspended with sterile PBS. The corresponding vehicle or 9CDHRA solution was carefully administered into the eyes through intravitreal injection (2 μL and 1 μM) following the steps described previously.^30^, ^31^ Mice were anesthetized in an induction chamber with 2–5% isoflurane in oxygen. During the procedure, anesthesia was maintained with 1–3% isoflurane in oxygen (0.6–1 L/min flow rate) while the animals were positioned on a warming pad to maintain body temperature. After ensuring complete anesthesia, the ocular surface was pretreated with 5% povidone‐iodine, followed by saline irrigation. Once the pupils were dilated with topical 1% tropicamide, topical anesthetic drops (Alcaine, proxymetacaine 0.5%) were applied before the injection. A single dose of 9CDHRA or vehicle solution (2 μL) was carefully administered through the sclera. The injection was performed at a 45° angle into the vitreous, directed toward the ora serrata and posterior to the temporal limbus, taking care to avoid contact with the lens. A 33G sterile, endotoxin‐free needle attached to a 5‐μL Hamilton syringe was used for the injection, which was performed under a surgical microscope (OPMI Vario S88, Carl Zeiss, Oberkochen, Germany) to ensure precise targeting. After injection, the needle was left in place for 10 s to allow diffusion of the solution and minimize reflux along the injection track. Post‐injection, 0.3% ciprofloxacin and 0.1% dexamethasone eye drops were applied. The intravitreal injection of vehicle or 9CDHRA solution was performed once weekly for 8 weeks. The animals were randomly allocated into four mice groups, each consisting of 10 mice: (1) control (normal IOP, vehicle‐treated) group, (2) control (normal IOP) group with 9CDHRA treatment, (3) high‐IOP‐subjected group (vehicle‐treated), and (4) high‐IOP‐subjected group with 9CDHRA treatment.

### 2.2 Chronic ocular hypertension animal model

To induce a chronic experimental model of glaucoma, mice were subjected to sustained elevation of intraocular pressure (IOP) through weekly intracameral injection of polystyrene microbeads (FluoSpheres polystyrene microspheres, 10 μm, Invitrogen, Thermo Fisher Scientific Pty Ltd) as described previously.^32^, ^33^ Briefly, mice were anesthetized with 2–5% isoflurane in oxygen and maintained on 1–3% isoflurane in oxygen (0.6–1 L/min flow of oxygen) on a heating pad during the injection procedure. After anesthesia, topical tropicamide 1% and proxymetacaine 0.5% (Alcaine, Alcon Laboratories NSW, Australia) eye drops were applied for pupil dilation and local anesthesia, respectively. Intracameral injections (2 μL; 3.6 × 10^6^ microbeads/mL) were performed using a Hamilton syringe connected to a disposable 33G needle (TSK Laboratory, Tochigi, Japan). The cornea was gently punctured near the limbus, and the needle was tangentially inserted beneath the corneal surface, releasing 2 μL of microbeads into the anterior chamber. All intraocular injection procedures were conducted under an operating microscope (OPMI Vario S88, Carl Zeiss, Oberkochen, Germany), ensuring no needle contact with the iris or lens. One eye was randomly selected for microbead injection, while the eyes of control (sham) animals (normal IOP) received an equivalent volume of sterile PBS. Weekly microbead injections were administered for the first 4 weeks to maintain IOP levels above 20 mmHg. If IOP declined after the fourth week, additional injections were administered during weeks five and six to sustain the target pressure. IOPs were measured using an iCare TonoLab rebound tonometer (iCare, Finland), designed for small animal models. Mice were anesthetized under isoflurane to minimize stress and ensure immobility during the procedure. The tonometer probe was positioned perpendicular to the central cornea at a distance of approximately 2–3 mm, avoiding contact with the corneal surface to prevent injury or artifact. The IOPs were measured weekly from four consecutive measurements obtained for each eye. Measurements were performed during the same time of day to account for diurnal variations in IOP. To maintain consistency, all measurements were conducted under identical environmental conditions, including lighting and temperature, to minimize variability. Proper calibration of the tonometer was confirmed before each use, following the manufacturer's instructions.^31^, ^34^

### 2.3 Electroretinographic (ERG) analysis

Scotopic electroretinographic recordings were conducted following established procedures using the Ganzfeld ERG system (Phoenix Research Laboratories, Pleasanton, CA, USA) as previously described.^31^, ^32^ Overnight dark‐adapted mice were anesthetized with intraperitoneal injections of ketamine and medetomidine (75 and 0.5 mg/kg, respectively) and placed on a warming pad to maintain body temperature. Pupils were dilated using 1% tropicamide, and topical anesthetic (Alcaine, Alcon Laboratories Pty Ltd) drops were applied under dim red light. The ground and reference electrodes were inserted into the tail and subcutaneously at the middle of the forehead, respectively. To maintain normal body temperature, an electric heating pad was employed throughout the recording process. The recording electrode (gold‐plate objective lens) was positioned on the corneal surface, applying carmellose for optimal cornea –electrode contact during ERG recordings. After achieving baseline stabilization in darkness, ERG responses were individually recorded from each eye. A dim stimulation (−4.3 log cd·s/m^2^) was delivered 30 times at a frequency of 0.5 Hz, and the measurement of the amplitude of the positive scotopic threshold response (pSTR) was conducted from the baseline to the approximately 120 ms positive peak.^34^ The pSTR response in full‐field ERG is often preferred due to its higher sensitivity to early signs of inner retinal dysfunction, as reported in previous studies.^35^, ^36^

### 2.4 Immunofluorescence staining

The immunofluorescence staining (IF) of retinal and optic nerve cryosections was performed using previously established protocols.^33^, ^34^ Enucleated mice eyes and optic nerve tissues were subjected to fixation for 2 h in freshly prepared 4% paraformaldehyde (PFA) and were subsequently washed three times with PBS to remove residual PFA. To maintain consistent orientation during tissue embedding, the eyeballs were marked with tissue marking dye. After the fixation process, eyes were immersed in a 30% sucrose solution overnight before being embedded in the optimal cutting temperature compound (OCT) (Sakura Finetek, CA, USA). Tissue sections, 7 μm thick for eyes and 5 μm for optic nerves, were prepared using a Leica CM1950 cryostat cryo section equipment (Leica Biosystems, Nussloch, Germany). The tissue sections were then treated with a blocking buffer containing 0.3% Triton X‐100 and 5% donkey serum (Sigma‐Aldrich, St. Louis, MO, USA) in PBS for 2 h at room temperature. After blocking, the tissue sections were subjected to overnight primary antibody incubation at 4°C prepared in antibody dilution buffer (1 × PBS/2% BSA/0.3% Triton X‐100). The following primary antibodies and their dilutions were used for IF staining: rabbit anti‐Iba1 (1:1500; Cat# 019‐19 741); rabbit anti‐GFAP (1:1500; Cat# Z0334); mouse anti‐phosphorylated neurofilament heavy chain (pNFH) (1:300; Cat# 801601) mouse anti‐acetylated lysine (1:500, Cat# MA1‐2021); mouse anti‐ABCA1 (1:1000; Cat# ab18180); rabbit anti‐RXRα (1:1000, Cat#3085) and mouse anti‐CHOP (1:300, Cat# 2895). Following primary antibody incubation, the tissue sections were washed three times with PBS and then incubated with the corresponding secondary antibodies (1:1000, donkey anti‐rabbit Cy3 or donkey anti‐mouse Alexa Fluor 488; Jackson ImmunoResearch Labs) for 60 min at room temperature. Following another round of PBS washings, the slides were mounted using anti‐fade mounting media with Prolong 4′,6‐diamidino‐2‐phenylindole (DAPI) (Life Technologies, Eugene, OR, USA). Image acquisition was performed using a Zeiss fluorescence microscope (ZEISS Axio Imager, Carl Zeiss, Oberkochen, Germany). Immunostaining of tissues from different mouse groups was performed under identical conditions and in parallel to minimize variability due to fixation, permeabilization, or staining procedures. To establish the specificity of the immunostaining, the antibodies were validated through negative controls (no primary antibody), IgG controls, and positive controls, which were processed in parallel (Figure S2). The images were captured within the dynamic range by adjusting the imaging parameters such as laser intensity, gain, and exposure time, and applying dynamic range calibration tools to prevent signal saturation. Once parameters were standardized, all images were captured under identical settings for comparison across treatment groups. Quantitative analysis of relative fluorescence intensity was performed using the ImageJ software (version 1.52; NIH, Bethesda, MD, USA) on the images obtained from three sections of each animal tissue. The images were converted to 8‐bit grayscale in ImageJ, and appropriate thresholding was applied to distinguish signal from background for accurate quantification. After setting a uniform threshold, relative mean immunofluorescence intensities were measured.^30^, ^33^

### 2.5 Histology

Following fixation, the eyes were processed in an automated Leica ASP200S tissue processor (Leica Biosystems) and were subsequently embedded in paraffin wax. To ensure consistent eye orientation, tissue marking dye was applied. Paraffin slices (7 μm thick) of retinal sections encompass the entire retina cut through the optic nerve head in a parasagittal plane containing both the superior and inferior retina within the optic nerve width. These sections were subjected to hematoxylin and eosin (H&E) staining using an optimized procedure.^31^, ^32^ Imaging was performed with a Zeiss Microscope (ZEISS Axio Imager Z2), and analysis was carried out using ImageJ. The number of cells in the ganglion cell layer (GCL) was quantified along a length of 500 μm (from 100 to 600 μm from the optic disc edge). Cell counts were derived from three consecutive sections of each animal eye and averaged to assess the GCL cell count (*n* = 4 animals/group).

### 2.6 Western Blot Analysis

Retinal tissues obtained from the eyes were immediately span frozen in liquid nitrogen and subsequently resuspended in ice‐cold lysis buffer (20 mM Tris–HCl, pH 8.0, 1% Triton X‐100, 2 mM EDTA, 2 mM PMSF, 100 mM NaCl, 1 mM Na3VO4, and complete protease inhibitor cocktail). Following homogenization by sonication on ice, the samples were centrifuged at 10 000 × *g* for 10 min at 4°C to collect protein supernatants. The protein concentration in the obtained lysates was determined using the Micro BCA assay (Thermo Fisher Scientific, MA, USA). Approximately 20 μg of proteins were resolved on SDS‐PAGE and electroblotted onto a nitrocellulose membrane using the Invitrogen iBlot2 system (Thermo Fisher Scientific, MA, USA) as previously described.^37^, ^38^, ^39^ Following protein transfer, the membranes were washed with TTBS (20 mM Tris–HCl pH 7.4, 100 mM NaCl, and 0.1% Tween 20) and blocked with BSA (3%) in TTBS for 1 h at room temperature. After that, primary antibody solutions were added, and the blot membranes were incubated overnight at 4°C. The following concentrations of primary antibodies were used: anti‐Iba1 (1:1000; Cat# 019–19 741), anti‐GFAP (1:1000; Cat# Z0334), anti‐Bcl2 (1:1000; Cat# 2876), anti‐Bad (1;1000; Cat# 9292), anti‐Bax (1:1000; Cat# ab32503), anti‐CHOP (1:1000, Cat# 2895), anti‐ATF4 (1:1000, Cat# 11815), anti‐ABCA1 (1:1000; Cat# ab18180), anti‐RXRα (1:1000, Cat#3085) and anti‐β‐actin (1:5000; Cat# ab6276). Following primary antibody incubation, membranes underwent four washes (5 min each) with TTBS and were subsequently incubated for 1 h at room temperature with secondary antibodies conjugated with horseradish peroxidase (anti‐rabbit 1:5000 and anti‐mouse 1:5000, Jackson ImmunoResearch Labs). After four additional washes, protein bands were visualized using enhanced chemiluminescence (Super Signal West Femto Maximum Sensitive Substrate; Thermo Fisher Scientific) following the manufacturer's instructions. Western blot images were captured with a Bio‐Rad ChemiDocMP Imaging system (Bio‐Rad Laboratories, Inc., Hercules, CA, USA). Mean densitometric analysis of protein band intensities was performed using ImageJ software, and the relative expression of target proteins was assessed after normalization to β‐actin.

### 2.7 Histone deacetylase (HDAC) enzyme activity assay

HDAC activity in retinal lysates from different mouse groups was measured using the fluorometric HDAC Activity Assay Kit (Abcam, #ab156064) following the manufacturer's protocol for a 96‐well plate format. The HDAC activity assay was conducted using a Pherastar microplate reader (BMG LABTECH, Germany) following the previously described procedure.^19^ Retinal lysates were prepared in an HDAC assay buffer, and protein concentration was determined using the BCA protein assay kit. For each well, 20 μg of retinal lysate was added, with blank wells containing assay buffer without lysate. HDAC substrate was then added to each well, ensuring thorough mixing with the lysates without introducing air bubbles. The plate was incubated for 30–60 min at room temperature, allowing HDAC enzymes to deacetylate the substrate and generate a fluorogenic product. Fluorescence intensity was measured using the Pherastar microplate reader with excitation at 360 nm and emission at 460 nm. Data were recorded over 90‐min time, and the changes in fluorescence intensity were plotted during the incubation intervals to assess HDAC activity.

### 2.8 Apoptotic assay (TUNEL staining)

To evaluate apoptotic changes in retinal cells, terminal deoxynucleotidyl transferase‐mediated dUTP‐biotin nick end labeling (TUNEL) staining was performed on paraffin‐embedded retinal sections using the DeadEnd Fluorometric TUNEL System kit (Promega), following a previously established procedure.^33^, ^40^ In brief, paraffin retinal sections were deparaffinized with xylene, rehydrated through decreasing ethanol concentrations, and subjected to a 15‐min fixation in a 4% paraformaldehyde (PFA) solution, followed by PBS washing. The rehydrated sections were permeabilized with proteinase K (20 μg/mL), washed, and refixed in 4% PFA for 5 min. After washing with PBS, the sections were incubated with a TUNEL reaction mixture (containing equilibration buffer, nucleotide mix, and rTdT enzyme) at 37°C for 1 h in the dark. To prevent tissue from drying and to ensure even distribution of the reaction mix, a plastic coverslip was placed over the slides during incubation. Following incubation, the slides (without the plastic coverslip) were immersed in 2× saline‐sodium citrate (SSC) buffer for 15 min to halt the reaction. Subsequently, the slides were washed with PBS and mounted with prolonged anti‐fade mounting media containing Prolong DAPI (Life Technologies, Eugene, OR, USA). The apoptotic cell staining was examined using epifluorescence microscopy (ZEISS Axio Imager, Carl Zeiss, Oberkochen, Germany), and TUNEL‐positive cells were quantified over a 600 μm length (in regions spanning 100–800 μm from the edge of the optic disk) on immunofluorescence images (n = four in each group).

### 2.9 Statistical analysis

All statistical analyses of STR amplitudes, histology, relative fluorescence intensity, western blot band density, and TUNEL assay data was performed using the GraphPad Prism 8 software (GraphPad Software Inc., San Diego, CA, USA). The sample size (*n*) in each figure indicates the number of tissues from the different animals of the same group. One‐way ANOVA was used to compare the groups statistically, and Tukey's test was used for multiple comparisons (GraphPad Prism). For each given *n* size, all data are shown as mean ± standard deviation of the mean (SD), and for data analysis, a *p*‐value <.05 was considered statistically significant.

## 3 RESULTS

### 3.1 9CDHRA treatment protects inner retinal function in glaucomatous injury

**FIGURE 1:**
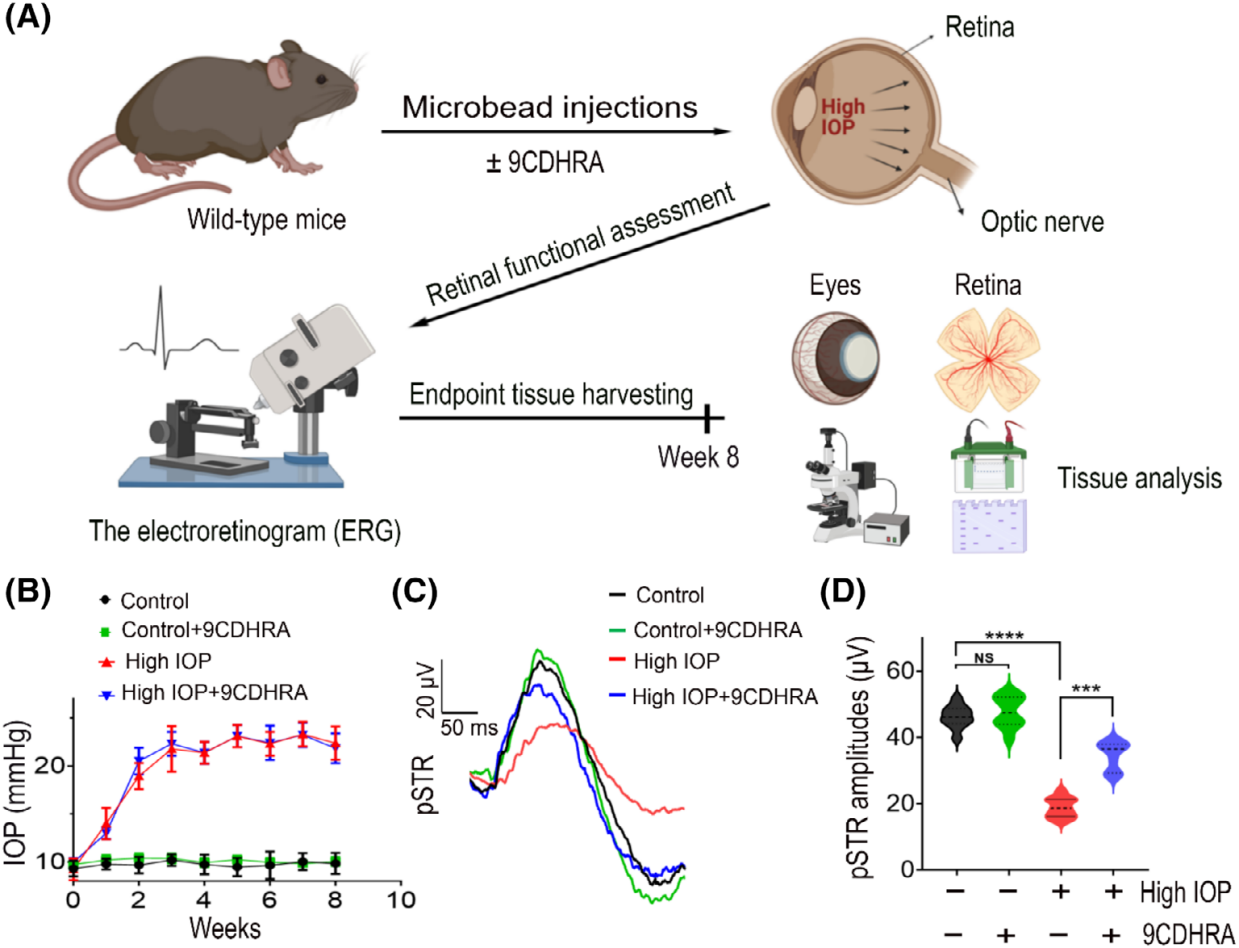
(A) Schematic diagram illustrating the experimental design and evaluation of retinal tissue for functionality, structure, and biochemical changes. (B) Chronic elevation of intraocular pressure (IOP) was induced in the eyes of wild‐type C57BL/6 mice through intracameral microbead injections for 8 weeks in different mouse groups, and the vehicle or 9CHDRA was administered via intravitreal injections. (C) Assessment of positive scotopic threshold responses (pSTR; averaged plots) in mouse eyes treated with or without 9CDHRA after 8 weeks of sustained elevated IOP. (D) Quantification of the pSTR amplitudes showing the significant protective effects of 9CDHRA on preserving the inner retinal function in high‐IOP induced glaucomatous injury conditions (NS, not significant, ****p < .0001, ***p < .001, one‐way ANOVA analysis with Tukey's multiple comparisons test, *n* = 10 per group).

The potential protective effects of 9CDHRA treatment on the retina against glaucomatous injury were evaluated using a chronic high‐IOP model in wild‐type C57BL/6 adult mice, as reported previously.^31^, ^33^ The eyes were subjected to intracameral microbead injections for 2 months to induce chronic elevation of IOPs, and the eyes received weekly vehicle or 9CDHRA administration through intravitreal injections. The dose of 1 μM used in this study was based on prior recommendations, as 9CDHRA binds to RXRs with high affinity to elicit downstream effects.^12^, ^14^ The retinal functional changes were analyzed using positive scotopic threshold response (pSTR) electrophysiological recordings before harvesting the tissues (Figure 1A). After receiving intracameral microbead injections, the mice groups showed gradually increased IOP to 20–25 mmHg. After 8 weeks, the IOPs of the mice treated with 9CDHRA were comparable to those of the vehicle‐treated control group (Figure 1B). Assessment of inner retinal function using pSTR amplitudes revealed a significant decrease in the high‐IOP‐subjected eyes, indicating a decline in the function of the inner retina compared to the normal IOP conditions. This high‐IOP‐induced loss of pSTR amplitudes was significantly protected in the eyes treated with 9CDHRA (*p* < .001; Figure 1C,D). The pSTR amplitude loss of 59.32 ± 3.01% observed in vehicle‐treated high‐IOP conditions was reduced to 22.21 ± 4.28% in the 9CDHRA treatment group. The pSTR amplitudes in the 9CDHRA‐treated mice were comparable to those in untreated mice under normal IOP conditions. These results suggested that RXR agonist 9CDHRA treatment in the retina is protective of neural function in glaucomatous injury conditions.

### 3.2 Treatment with 9CDHRA preserves retinal structure and optic nerve integrity against glaucomatous degeneration

**FIGURE 2:**
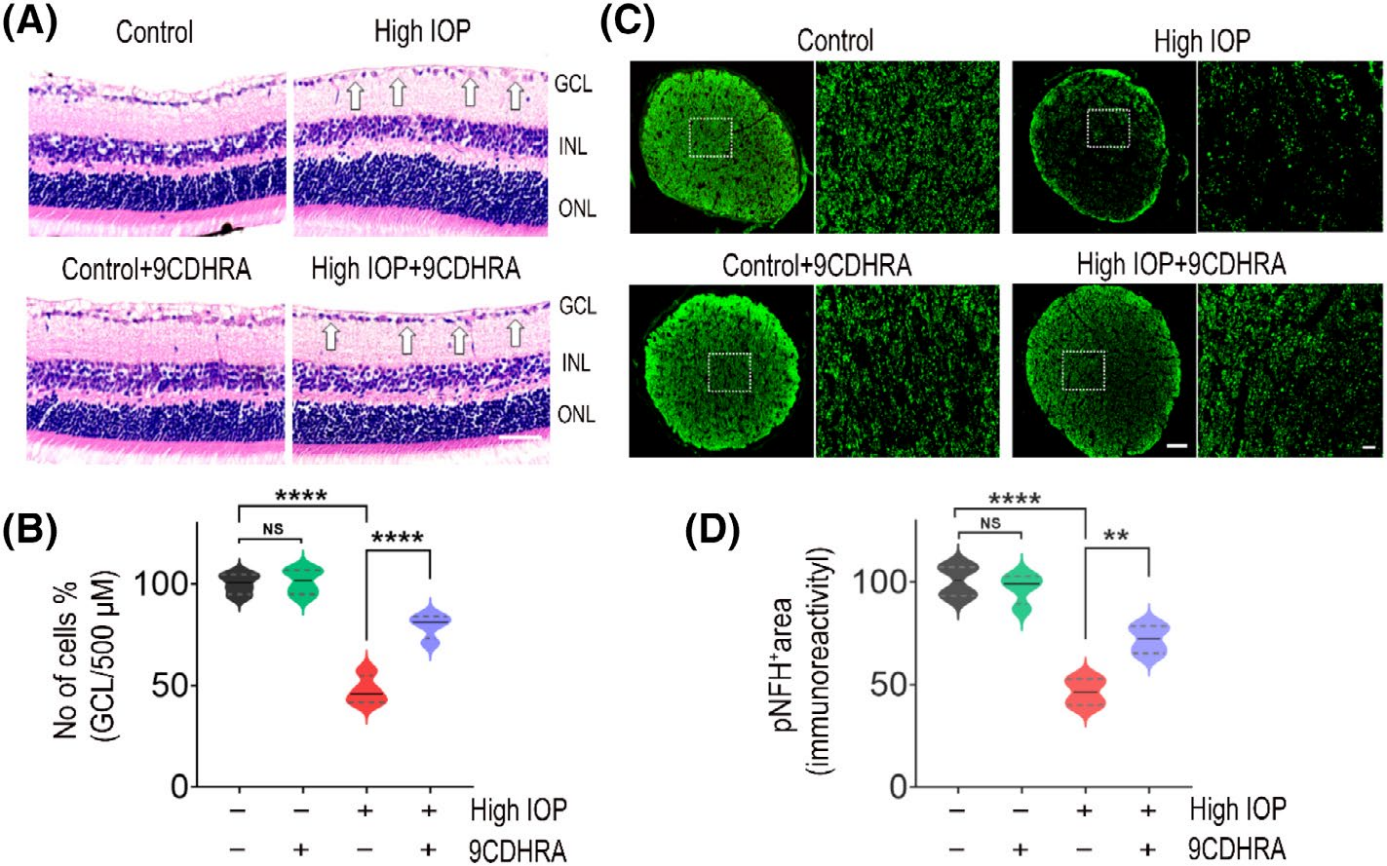
9CDHRA treatment protects inner retinal structure and optic nerve damage in chronic high‐IOP conditions. (A) Representative images of hematoxylin and eosin‐stained sagittal cross‐sections of the eyes, with arrows indicating changes in cell densities. Scale bar: 50 μm. Layers identified include the ganglion cell layer (GCL), inner nuclear layer (INL), and outer nuclear layer (ONL). (B) Quantification of cell density in the GCL from retinal hematoxylin and eosin‐stained images showing significant differences (NS, not significant, ****p < .0001, *n* = 4 per group). (C) Immunofluorescence staining of optic nerve cross‐sections using pNFH (SIM‐31), with green staining indicating pNFH+ areas. Scale bar: 50 μm for full sections and 10 μm for high magnification areas (boxed regions). (D) Quantification of immunoreactivity of pNFH in the optic nerves revealed significant differences, demonstrating the protective effects of 9CDHRA on the inner retinal structure and optic nerve under high‐IOP injury conditions. Statistical analysis was performed using one‐way ANOVA followed by Tukey's multiple comparisons test (NS, not significant, ****p < .0001, **p < .01, *n* = 4 per group).

We next evaluated the cellular density changes in the GCL and the extent of optic nerve (ON) damage by H&E histological analysis of retinal sections and immunofluorescence staining of optic nerves with phosphorylated neurofilament heavy chain (pNFH) antibody (Figure 2). The mice eyes subjected to microbead‐induced high IOP showed a significant loss of GCL cell density compared with the control normal IOP mice eyes (*p* < .0001). When compared to the vehicle‐treated mice group, the GCL cell density loss in the 9CDHRA‐treated mice group was significantly diminished under high‐IOP conditions. The GCL cell density decreased by 52.57 ± 7.08 in the vehicle‐treated mice group in high‐IOP conditions and was reduced to 20.58 ± 6.01% (*p* < .0001; Figure 2A,B) in the 9CDHRA‐treated mice group. The mice retinas treated with 9CDHRA showed similar GCL cell density under normal IOP conditions. Dephosphorylation of the neurofilament heavy chain in RGC axons has been observed in eyes with high IOP.^31^, ^41^ Hence, we used immunofluorescence labeling of ON cross‐sections with pNFH antibody to assess the degree of ON degeneration. Immunoreactivity measurements revealed a significant decrease in pNFH+ areas in high‐IOP ON sections. When the mice were treated with 9CDHRA, ON axonal damage observed in elevated IOP conditions decreased significantly from 53.59 ± 6.64% to 27.21 ± 6.92% (*p* < .01; Figure 2C,D). However, no significant changes were observed with 9CDHRA treatment under normal IOP conditions. These findings suggested that 9CDHRA has a protective effect on the retina and ON from neurodegenerative changes induced by chronically elevated IOP.

### 3.3 9CDHRA treatment attenuates ER stress responses in glaucoma conditions

**FIGURE 3:**
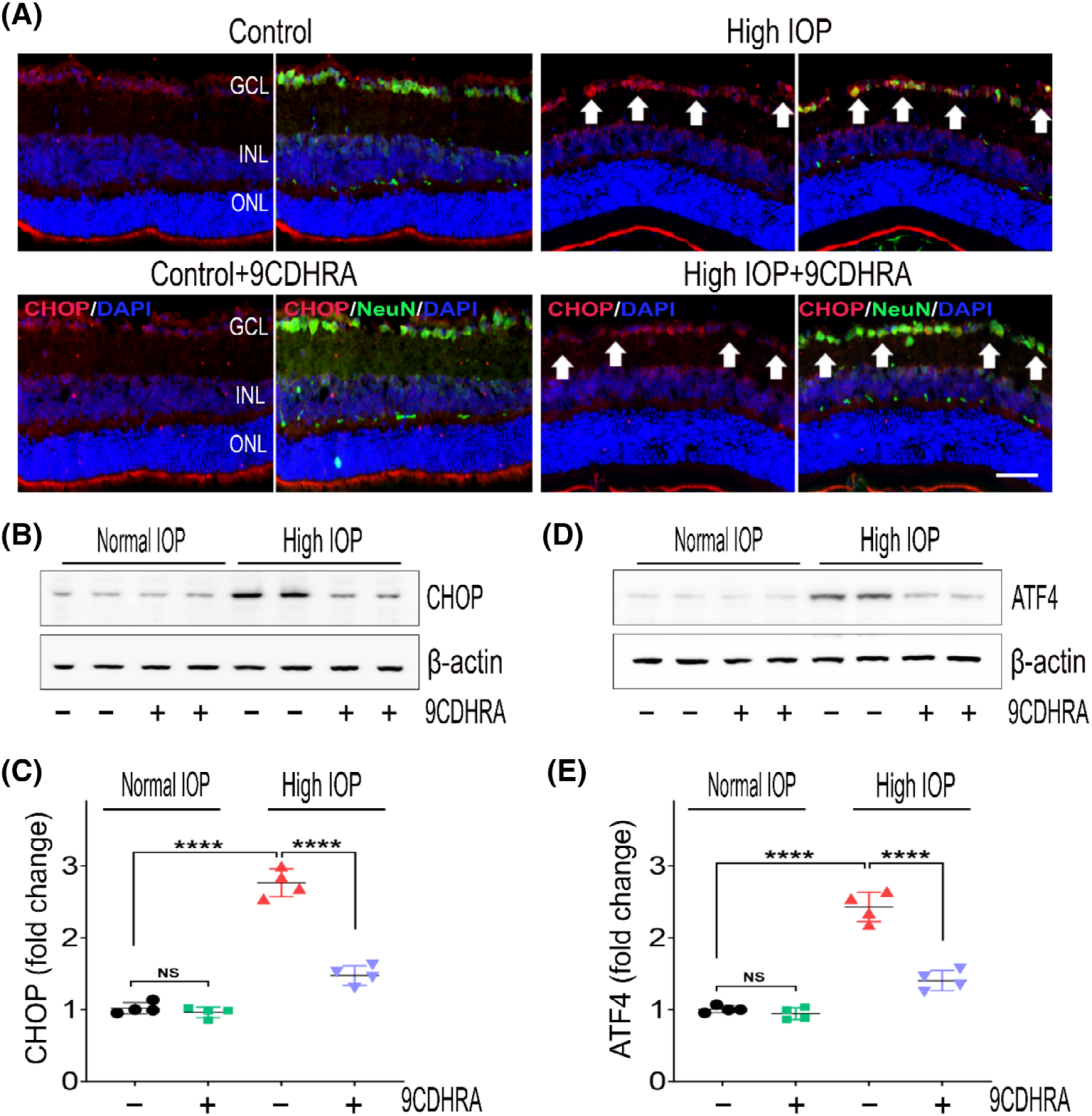
Impact of 9CDHRA treatment on the reduction of ER stress markers under chronic high‐IOP conditions. (A) Immunofluorescence images of eye sections stained with CHOP (red), the neuronal marker NeuN (green), and DAPI (blue) (representative images, scale bar: 50 μm; GCL, ganglion cell layer; INL, inner nuclear layer; ONL, outer nuclear layer), demonstrating the upregulation of CHOP and its modulation with 9CDHRA treatment in high‐IOP retinas. Arrows indicate the increase in CHOP immunoreactivity in glaucomatous injury compared to controls and its reduction following 9CDHRA treatment. (B) Representative western blots showing the expression levels of CHOP in retinal tissues under normal and high‐IOP conditions. (C) Densitometric analysis quantifying CHOP blot densities normalized to β‐actin. (D) Immunoblot analysis displaying ATF4 expression levels (representative blots) in retinal tissues. (E) Densitometric quantification of ATF4 band intensities normalized to β‐actin. Statistical analysis of the western blot results shows that CHOP and ATF4 protein levels significantly decreased with 9CDHRA treatment compared to the untreated group in high‐IOP injury conditions (NS, not significant, ****p < .0001, one‐way ANOVA with Tukey's multiple comparisons test, *n* = 4 per group).

The involvement of the ER stress response and activation of the unfolded protein response (UPR) pathway has been implicated in RGC degeneration in various experimental models of glaucoma.^24^, ^42^ Activation of RXRs by various ligands has been correlated with the modulation of UPR and other ER stress pathways.^2^, ^19^ Given these considerations, our next investigation focused on assessing the effects of 9CDHRA treatment on ER stress‐associated changes in the retina in glaucomatous conditions. The expression levels of ER stress‐associated marker proteins, specifically C/EBP homologous protein (CHOP) and activating transcription factor 4 (ATF4), were evaluated in retinal tissue samples using immunofluorescence staining and western blotting techniques. Immunofluorescence analysis of retinal sections using a CHOP antibody revealed enhanced reactivity, primarily within the GCL, exhibiting colocalization with the neuronal marker NeuN under elevated IOP conditions compared to ocular normotensive mice. Notably, this increased CHOP expression was markedly attenuated in mice treated with 9CDHRA in high‐IOP‐induced glaucomatous injury (Figure 3A). Additionally, western blot analysis of retinal lysates corroborated these findings, demonstrating a significant reduction in CHOP expression under high‐IOP conditions upon treatment with 9CDHRA. Western blot quantitative analysis demonstrated a 2.77 ± 0.20‐fold increase in CHOP expression in vehicle‐treated high‐IOP retinas, which was reduced to 1.47 ± 0.13‐fold in the 9CDHRA treatment group (*p* < .0001; Figure 3B,C). Similarly, a 2.43 ± 0.21‐fold increase in the expression of ATF4 observed in vehicle‐treated elevated IOP retinas was significantly reduced to 1.40 ± 0.12‐fold in 9CDHRA‐treated mice retinas (*p* < .0001; Figure 3D,E). No significant changes were noticed in the expression of these proteins in the retinal samples under normal IOP conditions. These results suggested that 9CDHRA treatment is involved in suppressing the ER stress pathway activation in the retina in glaucomatous conditions.

### 3.4 Suppression of HDAC activity with 9CDHRA treatment

**FIGURE 4:**
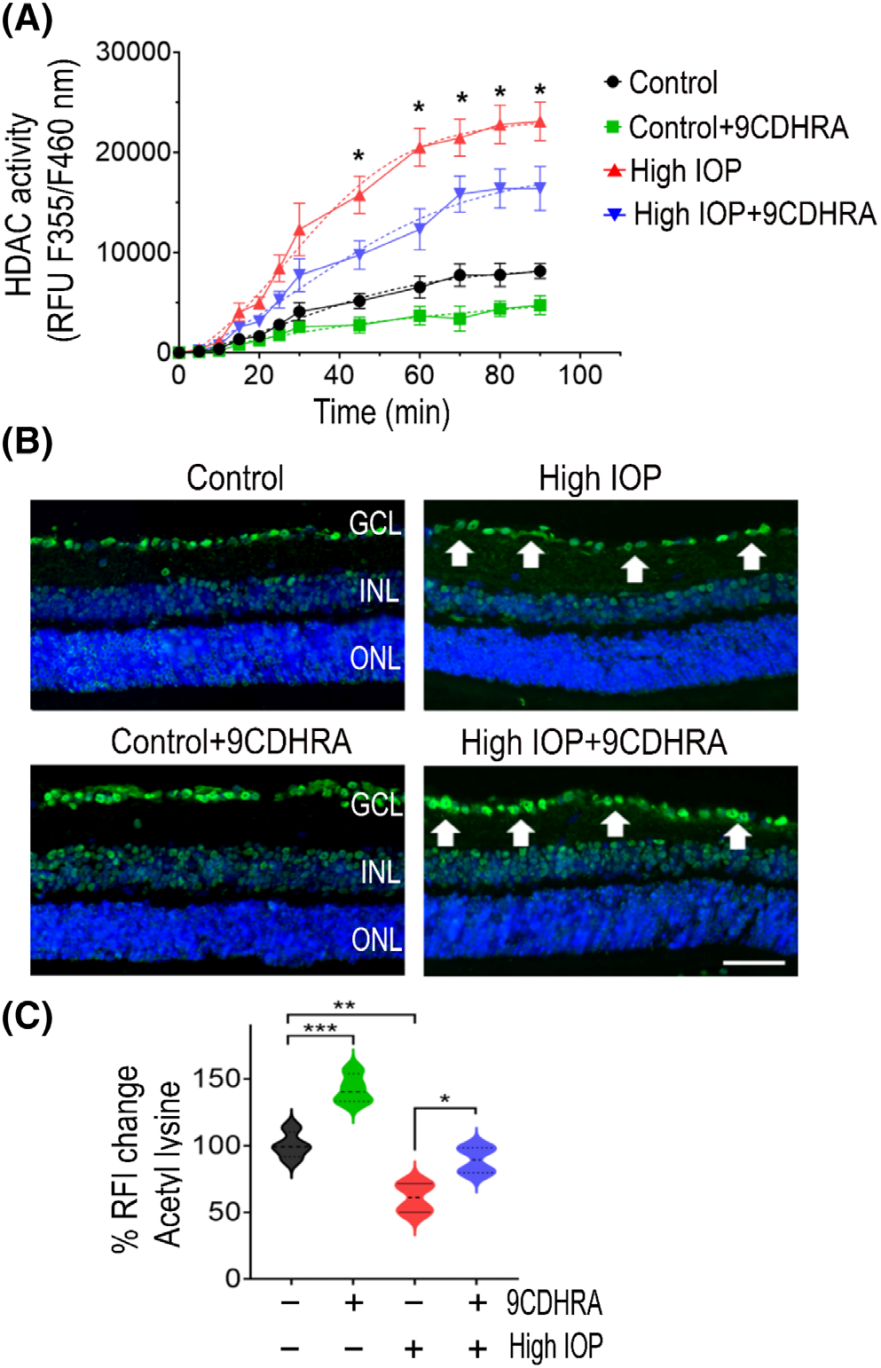
Effects of 9CDHRA treatment on HDAC inhibition and protein lysine acetylation. (A) Time‐dependent HDAC enzyme activity was measured in retinal tissue lysates from different groups of mice. The resulting data is plotted on a graph showing an increased HDAC activity in high‐IOP retinas, which decreases with 9CDHRA treatment. The dotted lines represent non‐linear regression least squares Sigmoidal fit (*p < .05). (B) Immunofluorescence images of the eye sections stained with acetyl lysine antibody (green) and DAPI (blue) (representative images: Scale bar, 50 μm; GCL, ganglion cell layer; INL, inner nuclear layer; ONL, outer nuclear layer), arrows indicate the changes in acetylated lysine staining in the GCL. (C) Quantitative immunoreactivity measurements reveal a significant upregulation of protein acetylation with 9CDHRA treatment (*p < .05, **p < .01, ***p < .001, one‐way ANOVA analysis with Tukey's multiple comparisons test; *n* = 4 per group).

RXR activation and histone deacetylase (HDAC) regulation are two interconnected processes governing gene expression and diverse cellular functions.^43^, ^44^ RXR activation has been shown to have an inverse correlation with HDAC regulation, and increased HDAC activity has been implicated as playing an important role in inducing RGC degeneration and ON damage in retinal degradative conditions.^45^, ^46^, ^47^ To explore the effects of 9CDHRA on HDAC activity, we analyzed its activity in retinal tissue samples using a fluorometric activity assay kit. Similar to earlier studies on HDAC activation in RGC damage models, we found elevated HDAC activity in high‐IOP conditions. This increased HDAC activity seen in glaucomatous retinas was significantly reduced in mice eyes treated with 9CDHRA (*p* < .05; Figure 4A). In a previous study, suppression of HDAC activity in elevated IOP conditions increased protein acetylation, leading to protective effects against RGC loss.^48^ We aimed to assess the protein acetylation changes in the retina by immunofluorescence staining using the antibody against acetylated lysine 9 of histone H3. In the retinas of mice with normal IOP, lysine acetylation staining was observed throughout the inner nuclear layer and ganglion cell layer. Conversely, the retinas of mice subjected to high IOP exhibited markedly diminished lysine acetylation in the ganglion cell layer. However, mice eyes treated with 9CDHRA showed increased acetylated lysine staining in the retina compared to the vehicle‐treated mice group. Quantitative immunoreactivity measurements showed significantly increased lysine acetylation (*p* < .001; Figure 4B,C) with 9CDHRA treatment in high‐IOP conditions. Taken together, these results suggest that 9CDHRA treatment was associated with negative regulation of HDAC activity that correlated with increased protein acetylation in the retina in glaucoma conditions.

### 3.5 Reduced apoptotic pathway activation in glaucoma upon 9CDHRA treatment

**FIGURE 5:**
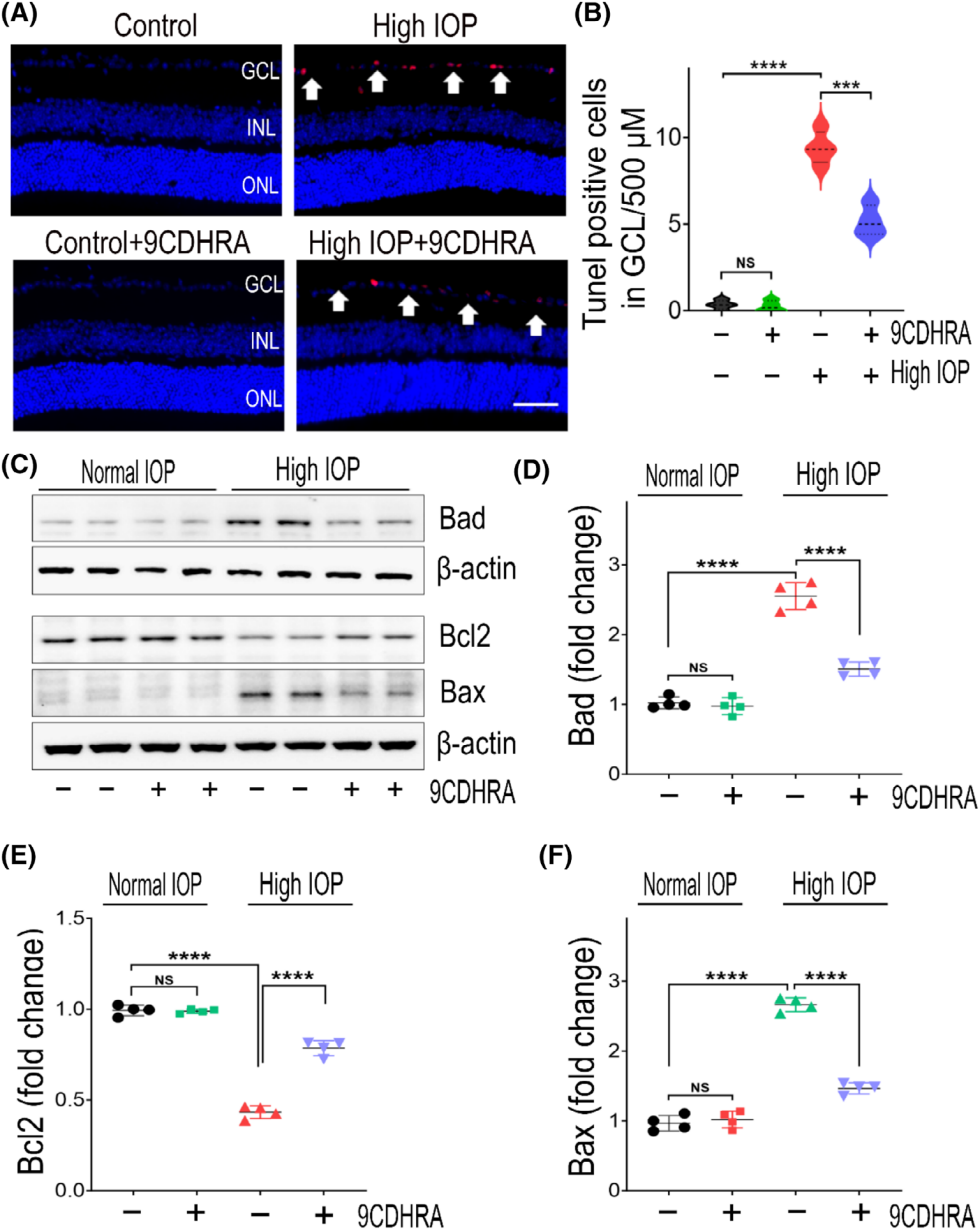
Eyes treated with 9CDHRA exhibit reduced apoptotic changes in the ganglion cell layer under experimental glaucoma conditions. (A) Assessment of TUNEL apoptotic changes conducted in the 100–700 μM region from the edge of the optic disk in retinal sections. The arrows indicate changes in TUNEL staining in the ganglion cell layer after 8 weeks of elevated IOP induction. The representative image shows TUNEL staining (red) and nuclear staining with DAPI (blue) (Scale bar = 50 μM. GCL, ganglion cell layer; INL, inner nuclear layer; ONL, outer nuclear layer). (B) Quantification of TUNEL‐positive cells in the GCL of retinas revealed a significant decrease in the number of TUNEL‐positive cells in the retinas treated with 9CDHRA under high‐IOP conditions (NS, not significant, ***p < .001, ****p < .0001, one‐way ANOVA with Tukey's multiple comparisons test, *n* = 4 per group). (C) Representative immunoblots displaying the expression levels of the apoptotic pathway proteins Bad, Bcl2, and Bax. The band intensities from the western blots were quantified densitometrically and normalized to β‐Actin for (D) Bad expression, (E) Bcl2 expression, and (F) Bax expression. Treatment with 9CDHRA significantly restored the changes in apoptotic markers under high‐IOP conditions (NS, not significant, ****p < .0001, one‐way ANOVA with Tukey's multiple comparisons test, *n* = 4 per group).

In glaucomatous conditions, RGCs have been demonstrated to undergo degeneration and cell death primarily through the apoptotic pathways.^24^, ^49^ We examined the apoptotic changes in retinal sections to investigate the neuroprotective impact of 9CDHRA in glaucomatous conditions. The retinas assessed by TUNEL assay showed significantly increased apoptotic cells in the ganglion cell layer (*p* < .0001) under chronic high IOP conditions. However, treatment with 9CDHRA significantly mitigated the presence of TUNEL‐positive cells in high‐IOP retinas (Figure 5A,B). Next, we assessed alterations in the expression levels of apoptotic pathway proteins, including Bax, Bcl2, and Bad, within retinal lysates using western blot analysis (Figure 5C). These proteins belong to the Bcl2 family, where Bcl‐2 functions as an antiapoptotic factor, while Bax and Bad act as proapoptotic factors.^50^ These proteins are crucial in controlling apoptosis, and their expression changes have been observed in the RGCs in response to glaucomatous injury.^51^ Western blot densitometric quantitative analysis of retinal lysates revealed a significant decrease in the expression of the antiapoptotic protein Bcl‐2, which was reduced by 1.75 ± 0.19‐fold, while the proapoptotic proteins Bax and Bad showed a significant increase of 2.66 ± 0.11‐fold and 2.55 ± 0.20‐fold, respectively, under high‐IOP conditions in the vehicle‐treated mice group. The 9CDHRA treatment restored these expression changes to a significant extent in the high IOP‐exposed mice group. The 9CDHRA‐treated mice showed significantly increased Bcl2 expression and suppressed the proapoptotic Bax and Bad in high‐IOP glaucomatous injury (*p* < .0001; Figure 5D–F). No significant changes were observed in the expression of these apoptotic pathway proteins in the retinal samples treated with 9CDHRA under normal IOP conditions. Collectively, these results suggested that RXR agonist 9CDHRA treatment suppressed the proapoptotic pathway in experimental glaucomatous injury conditions.

### 3.6 Induction of RXR‐regulated ABCA1 expression with 9CDHRA treatment in the retina

**FIGURE 6:**
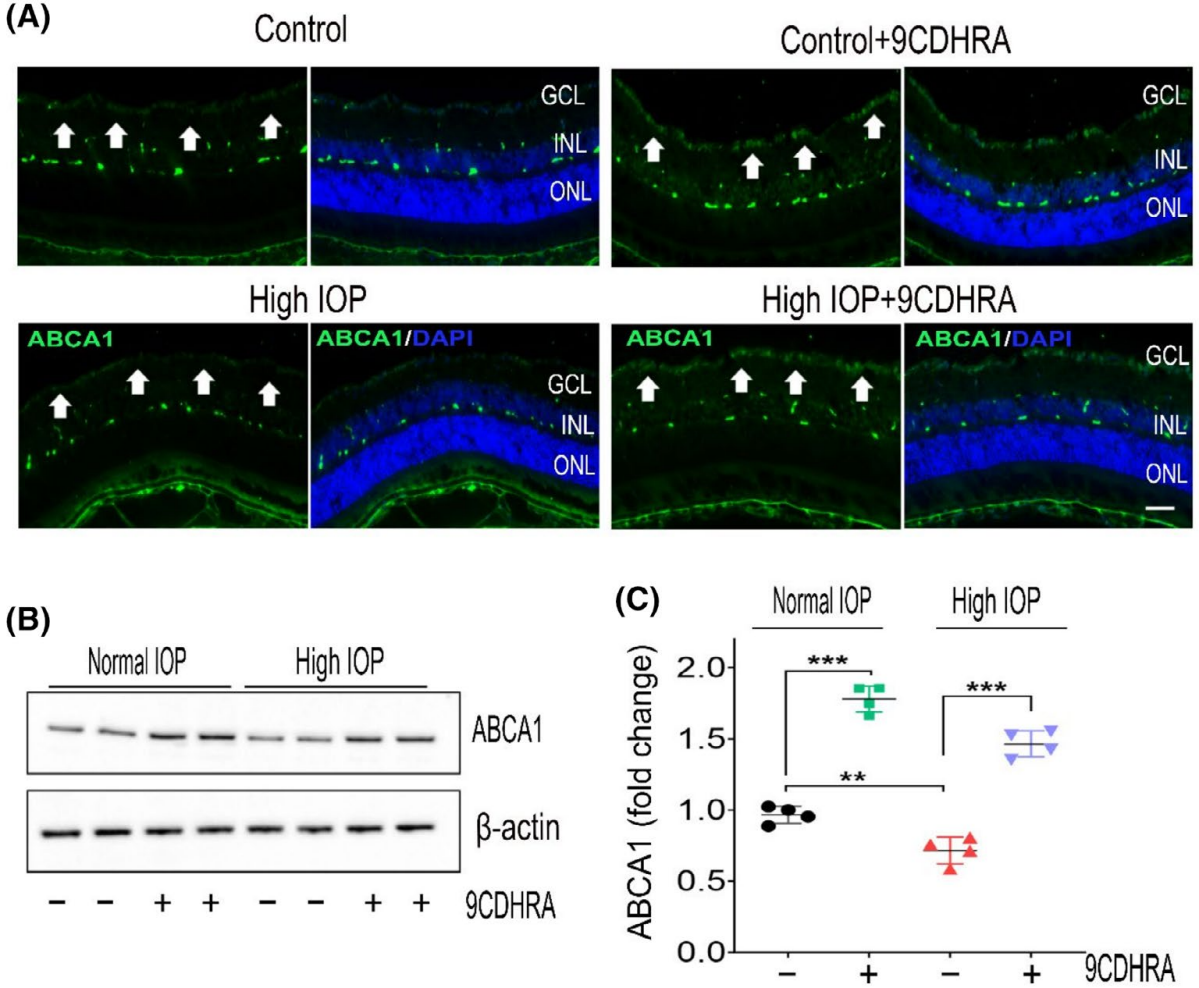
Upregulation of ABCA1 expression in the retinas treated with 9CDHRA. (A) Immunofluorescence images of retinal sections stained for ABCA1 (green) and DAPI (blue) (representative images, scale bar: 50 μm; ONL, outer nuclear layer; INL, inner nuclear layer; GCL, ganglion cell layer). ABCA1 expression is upregulated in the GCL with 9CDHRA treatment (arrows indicate changes in ABCA1 immunoreactivity). (B) Western blot analysis of retinal tissues showing ABCA1 protein levels (representative blots). (C) Densitometric quantification of ABCA1 expression (normalized to β‐actin) reveals a significant decrease in ABCA1 levels in high‐IOP retinas, with its upregulation following 9CDHRA treatment under both normal and high‐IOP conditions (**p < .01, ***p < .001, one‐way ANOVA with Tukey's multiple comparisons test, *n* = 4 per group).

Activation of RXRs by agonists has been shown to upregulate ATP‐binding cassette subfamily A member 1 (ABCA1) expression through heterodimerization with LXRs in several studies.^52^, ^53^, ^54^ ABCA1 is expressed in the retina, including RGCs, and its reduced expression is considered a risk factor for glaucoma.^55^, ^56^ To determine whether RXR activation via 9CDHRA treatment induces ABCA1 expression, we analyzed retinal tissues using immunostaining and western blotting. We first evaluated the effects of 9CDHRA treatment on RXRα expression in the retina. Our previous studies have documented RXR expression in RGCs and its downregulation under glaucomatous conditions.^19^ Consistent with prior findings, immunofluorescence staining of retinal sections revealed decreased RXR expression in the GCL of glaucomatous mice compared to the control mice group (Figure S1A). However, this reduction was markedly preserved in the 9CDHRA‐treated group. Quantitative immunoblot analysis of retinal lysates confirmed a significant downregulation of RXR in high‐IOP mice retinas compared to controls. Notably, 9CDHRA treatment effectively prevented RXR downregulation in glaucomatous mice retinas (Figure S1B, C). Next, we assessed ABCA1 expression, and our immunofluorescence staining of retinal sections showed a marked increase in ABCA1 expression in the GCL layer of 9CDHRA‐treated mice (Figure 6A). Similarly, immunoblotting analysis of retinal lysates demonstrated a significant decrease in ABCA1 expression under high‐IOP conditions, whereas 9CDHRA‐treated mice exhibited a significant upregulation of ABCA1 in both normal and high‐IOP conditions (Figure 6B,C), consistent with the immunofluorescence results. These findings suggest that RXR activation via 9CDHRA treatment induces ABCA1 expression in the retina, contributing to protective effects against glaucomatous injury.

### 3.7 9CDHRA treatment reduces glial activation in glaucomatous injury

**FIGURE 7:**
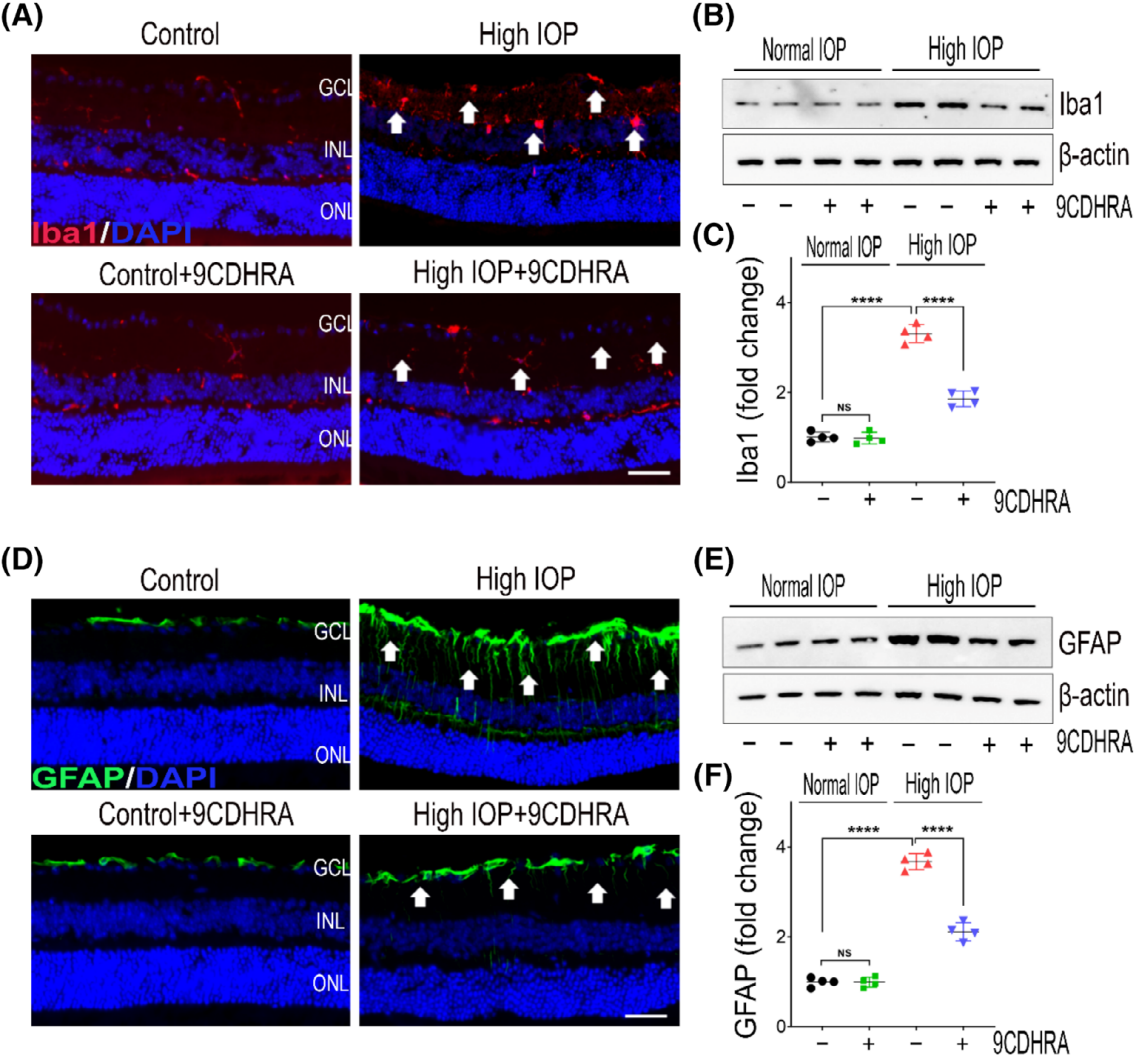
Suppression of retinal glial activation with 9CDHRA under experimental glaucoma conditions. (A) Immunofluorescence images of eye sections stained with Iba1 (red) for microglia and DAPI (blue) (representative images, scale bar: 50 μm; ONL, outer nuclear layer; INL, inner nuclear layer; GCL, ganglion cell layer) demonstrate microglial activation and its modulation with 9CDHRA treatment in high‐IOP retinas (arrows indicate changes in Iba1 immunoreactivity). (B) Western blot analysis of retinal tissues for Iba1 levels following high‐IOP injury (representative blots). (C) Densitometric quantitative analysis of Iba1 blot densities (normalized to β‐actin) reveals increased Iba1 levels in high‐IOP retinas and a significant reduction with 9CDHRA treatment (NS, not significant, ****p < .0001, one‐way ANOVA with Tukey's multiple comparisons test, *n* = 4 per group). (D) Immunofluorescence images of eye sections stained with GFAP (green) and DAPI (blue) for analysis of reactive gliosis (*Müller glia*) (representative images, scale bar: 50 μm; GFAP expression and hypertrophy changes indicated by arrows). (E) Western blot analysis of GFAP protein expression in retinal lysates (representative blots). (F) Densitometric quantitative analysis of GFAP blots shows a significant reduction in GFAP expression with 9CDHRA treatment under high‐IOP injury conditions (NS, not significant, ****p < .0001, one‐way ANOVA with Tukey's multiple comparisons test, *n* = 4 per group).

**FIGURE 8:**
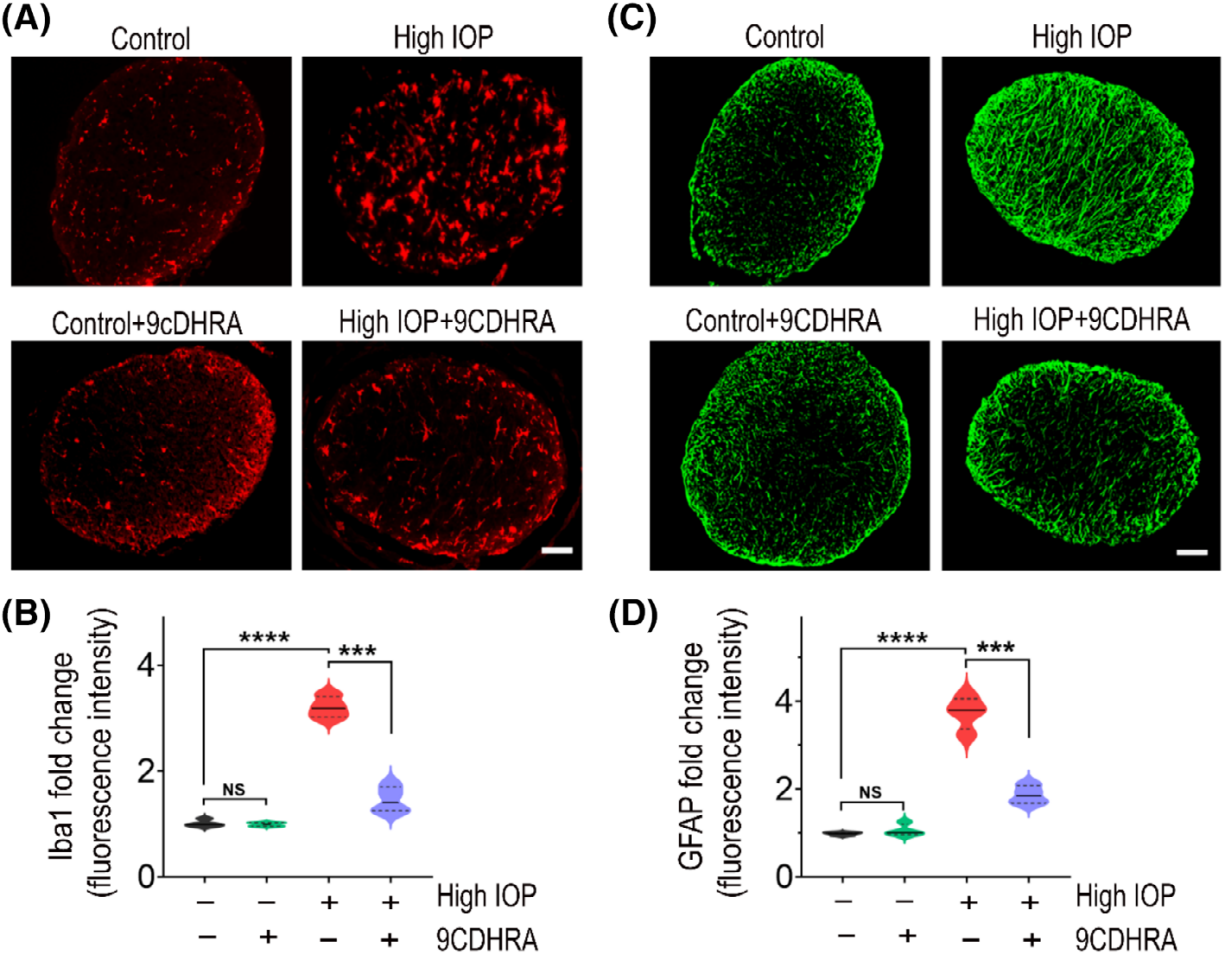
9CDHRA treatment attenuates glial activation in the optic nerves in experimental glaucoma conditions. (A) Immunofluorescence images of optic nerve cross‐sections (representative, scale bar: 50 μm) stained for microglia with Iba1 (red). (B) Quantification of Iba1 immunoreactivity indicating the microglia activation induced by high IOP was significantly reduced following 9CDHRA treatment (NS, not significant, ***p < .001, ****p < .0001, one‐way ANOVA with Tukey's multiple comparisons test, *n* = 4 optic nerves per group). (C) Representative immunofluorescence images of optic nerve cross‐sections stained for reactive astrogliosis with GFAP (green), scale bar: 50 μm. (D) Quantitative analysis of GFAP immunoreactivity demonstrating 9CDHRA treatment significantly suppressed astrogliosis in experimental glaucomatous injury (NS, not significant, ***p < .001, ****p < .0001, one‐way ANOVA with Tukey's multiple comparisons test, *n* = 4 per group).

Chronic glaucoma conditions are greatly influenced by neuroinflammatory glial cell activation, which has been shown to play an important role in disease progression.^57^, ^58^ To examine this phenomenon, we assessed glial activation in both retinal and ON tissues of mice eyes, utilizing Iba1 as a marker for microglia and GFAP for Müller glia and astrocytes. Immunofluorescence analysis of retinal sections indicated a markedly elevated presence of Iba1‐positive cells with activated morphology in response to high‐IOP injury compared to control mice retinas. Notably, this activation was mitigated in the retinas treated with 9CDHRA (Figure 7A). Further, densitometric quantitative analysis of western blot bands revealed significantly increased levels (3.31 ± 0.21‐fold) of Iba1 expression in the high‐IOP group retinal lysates vehicle‐treated compared to controls, and this increased Iba1 expression was significantly reduced in the mice retinas treated with 9CDHRA (*p* < .0001; Figure 7B,C). Similar to the retina, ONs exhibited microglial activation in response to high‐IOP injury. Quantitative measurements of Iba1 immunoreactivity revealed a significant decrease in microglial activation (*p* < .001; Figure 8A,B) following 9CDHRA treatment under high‐IOP conditions. Iba1 expression in the 9CDHRA‐treated retinas was similar to that in the control, untreated retinal samples under normal IOP conditions. We assessed reactive gliosis by examining the expression of the intermediate filament protein GFAP. In control normal IOP animals, GFAP immunoreactivity was localized to the GCL in retinal cross‐sections. However, in retinas subjected to elevated IOP, GFAP labeling was increased, with Müller cell processes extending into the inner nuclear layer (Figure 7D). Similarly, hypertrophic reactive astrogliosis, characterized by GFAP upregulation, was evident in ON sections under high‐IOP conditions (Figure 8C). Treatment with 9CDHRA resulted in reduced reactive gliosis both in the retina and ONs. Quantitative analysis of retinal lysates via western blotting revealed a 3.68 ± 0.18‐fold upregulation of GFAP expression in high‐IOP retinas compared to the normal IOP group. However, 9CDHRA treatment significantly suppressed GFAP expression compared to vehicle‐treated mice subjected to high‐IOP injury (*p* < .0001; Figure 7E,F). Furthermore, the increased GFAP immunoreactivity observed in optic nerves under high‐IOP injury was significantly reduced (*p* < .001; Figure 8D) in 9CDHRA‐treated mice. No significant differences were found between 9CDHRA‐treated and untreated control retinas under normal IOP conditions. In summary, these findings suggest that 9CDHRA treatment contributes to the suppression of glial activation in response to high‐IOP glaucomatous injury.

## 4 DISCUSSION

As ligand‐activated transcription factors, retinoid X receptors (RXRs) play crucial roles in the intricate molecular networks that govern CNS function and dysfunction. RXRs are nuclear receptors that regulate the expression of specific genes in response to binding with ligands. A variety of compounds, including retinoids and fatty acids, have been suggested as natural binding partners for RXRs.^2^, ^6^, ^7^ However, 9‐cis‐13,14‐dihydroretinoic acid (9CDHRA) has recently gained attention as an endogenous RXR ligand, sparking a growing interest in its potential roles.^10^, ^14^ Importantly, 9CDHRA is an active molecule that belongs to the vitamin A5/X class, a novel category of vitamin A. This class includes 9‐cis‐13,14‐dihydroretinol and 9‐cis‐13,14‐dihydro‐β, β‐carotene as nutritional precursors, distinguishing them from the pathways associated with classical vitamin A signaling.^6^, ^12^ In this study, we provided strong evidence for the neuroprotective benefits of 9CDHRA treatment in a mouse model of high‐IOP‐induced glaucoma. The study comprehensively assessed the impact of 9CDHRA treatment on retinal function, structure, including ER stress, HDAC activity, apoptotic pathway activation, and glial activation in glaucomatous conditions, demonstrating neuroprotective findings.

In the retina, RXRs are involved in the regulation of gene expression related to retinal development, cell differentiation, and maintenance of retinal integrity.^59^ Previously, the activation of RXRs has been shown to promote the survival of photoreceptors via inhibiting apoptosis in a mouse model of retinitis pigmentosa.^27^ We have previously reported that the expression of RXRs is negatively affected in the glaucomatous retinas, and we observed that the pharmacological agonist of RXR, bexarotene, elicited protective effects against the onset of glaucomatous conditions.^19^ Firstly, our results in this study demonstrated that 9CDHRA treatment protects inner retinal function against high‐IOP glaucomatous injury. By employing positive scotopic threshold response (pSTR) electrophysiological recordings, we found significant preservation of pSTR amplitudes in eyes treated with 9CDHRA compared to vehicle‐treated high‐IOP‐induced glaucomatous eyes. The structural integrity of the retina and ON, evaluated by histological and immunofluorescence analysis, demonstrated a significant attenuation of GCL cell density loss and ON axonal damage in 9CDHRA‐treated eyes compared to vehicle‐treated glaucomatous eyes. These findings suggest that 9CDHRA treatment maintains inner retinal function and preserves retinal and ON structure, indicating its potential to mitigate neurodegenerative changes associated with glaucoma.

Next, we investigated the molecular mechanisms underlying the neuroprotective effects of 9CDHRA treatment. In glaucoma, ER stress may play a significant role in disease development and progression.^60^ The activation of the ER stress pathway has been extensively documented in the retina of glaucoma mouse models, underscoring its pivotal role in the degeneration of RGCs.^60^, ^61^, ^62^ One intriguing aspect of RXR activation is that it plays a critical role in mitigating ER stress and maintaining cellular equilibrium via modulating various cellular processes.^29^, ^63^ Our findings indicate that 9CDHRA treatment mitigated ER stress responses in glaucomatous conditions, as evidenced by the assessment of ER stress‐associated marker proteins. Through immunofluorescence and western blot analyses, we observed a significant decrease in the expression levels of key ER stress markers, including CHOP and ATF4, with 9CDHRA treatment compared to vehicle‐treated glaucomatous retinas. 9CDHRA treatment alleviated ER stress to promote RGC survival in glaucomatous injury. Previous studies have also demonstrated that the activation of RXRs by pharmacological agonists attenuated the expression of unfolded protein response target genes, including CHOP, thereby reducing ER stress‐induced apoptosis in cellular stress environments including neurodegenerative conditions.^19^, ^29^, ^63^

Furthermore, this study investigated the role of histone deacetylase (HDAC) activity in mediating the neuroprotective effects of 9CDHRA treatment. HDAC and RXR have been proposed to mutually influence each other's functions, and the activation of RXRs was demonstrated to trigger transcriptional repression by impacting HDAC activity.^2^, ^43^, ^44^, ^64^ Previous studies have shown a correlation between the activation of cellular apoptotic pathways and epigenetic mechanisms, including HDAC activation in RGCs.^65^, ^66^ The expression of class 1 HDAC enzyme in the retina, notably within the GCL, and inhibition of HDAC in the retina via pharmacological approaches or targeted removal have been shown to confer protection to RGCs against injury from optic nerve crush.^45^, ^67^, ^68^ Our results revealed that elevated HDAC activity observed in glaucomatous retinas was significantly reduced following 9CDHRA treatment. In addition, decreased protein acetylation observed specifically in the ganglion cell layer in the high‐IOP retinas was preserved with 9CDHRA treatment that positively correlated with HDAC inhibition. In order to understand how these intracellular biochemical network changes impact RGC survival, we explored the apoptotic pathway in the retinas. TUNEL assay and western blot analysis revealed a significant increase in apoptotic cell death and imbalanced levels of antiapoptotic (Bcl2) and proapoptotic (Bax, Bad) proteins in glaucomatous retinas. These proapoptotic changes were significantly suppressed in 9CDHRA‐treated retinas. These findings indicated that 9CDHRA treatment attenuated apoptotic pathway activation via modulation of altered molecular networks, thereby promoting RGC survival in glaucomatous injury.

Our findings demonstrate that activation of RXRs by 9CDHRA treatment significantly induces ABCA1 expression in the retina. These results highlight the neuroprotective potential of RXR agonists in regulating lipid homeostasis and cellular survival in glaucoma. ABCA1, a key target of RXR‐LXR signaling, is essential for lipid transport, cholesterol homeostasis, and cell membrane integrity, which are critical factors for neuronal function and survival.^52^, ^69^ Previous studies have indicated that reduced ABCA1 expression is associated with increased susceptibility to neurodegenerative diseases, including glaucoma.^55^, ^69^, ^70^ Mice with ABCA1 deficiency exhibit elevated retinal cholesterol levels and progressive RGC loss with aging.^55^ Furthermore, ABCA1 dysfunction can impair lipid metabolism and exacerbate oxidative stress and inflammation.^70^, ^71^ In this study, we observed significant downregulation of ABCA1 in the high‐IOP group. The upregulation of ABCA1 following 9CDHRA treatment suggests that RXR activation may counteract these pathological mechanisms, thereby supporting RGC survival and function.

We also investigated the impact of 9CDHRA treatment on glial activation in the glaucomatous retinas. Retinal microglia and macroglia (astrocytes and Müller glial) serve as the central components of the innate immune system, and their early activation has been linked to neuronal damage in glaucoma.^57^, ^58^ Glial cell activity analyzed by immunofluorescence and western blotting on the retina and optic nerve tissues revealed their activation in terms of morphology and upregulation of Iba1 and GFAP in response to high‐IOP‐induced injury. A significant reduction was observed in microglial activation and reactive gliosis both in the retinas and optic nerves following 9CDHRA treatment. RXR heterodimerization with other nuclear family receptors such as RARs, PPAR, LXR, and Nurr1 plays important roles in regulating the adaptive immune response of immune cells via various processes, including clearing cellular debris, facilitating cell differentiation, and controlling the repression of genes encoding inflammatory mediators.^72^, ^73^, ^74^, ^75^ Attenuation of inflammation upon RXR activation has been reported previously in various studies.^76^, ^77^, ^78^ These findings indicated that 9CDHRA treatment suppressed neuroinflammatory responses associated with glaucomatous injury, further supporting its potential as a neuroprotective agent against glaucoma.

The neuroprotective effects observed with RXR activation following 9CDHRA treatment in this study are likely to influence other nuclear receptor partners. Importantly, RXRs form functional heterodimers with RARs in the retina, where they play key roles in regulating cellular differentiation, apoptosis, and neuroinflammation processes critical for retinal RGC survival in glaucoma.^2^, ^79^, ^80^, ^81^ Although 9CDHRA is a selective RXR agonist, RXR activation may indirectly overlap with RAR‐mediated pathways due to the intrinsic functional synergy between these receptor families. Future studies are required to explore the crosstalk between RXR and RAR signaling to better understand their combined influence on glaucomatous neurodegeneration. In summary, this study provides comprehensive evidence for the neuroprotective effects of 9CDHRA treatment in a mouse model of glaucoma. By preserving retinal function and structure, suppressing ER stress and HDAC activity, attenuating apoptotic pathway activation, and reducing glial activation, 9CDHRA may serve as a novel and promising therapeutic candidate in glaucoma management.

## AUTHOR CONTRIBUTIONS

D.B., N.C., V.G., W.K., V.B.G., and S.L.G. designed the research; D.B., N.C., S.S.O.M., V.P., G.E.P., A.S., A.M.K., and M.M. participated in performing experiments and analyzing data; All authors participated in writing and reviewing the manuscript.

## DISCLOSURES

W.K. is a shareholder of CisCarex U.G. Other authors declare that they have no competing interests.

## Supporting information


Figure S1.

## Data Availability

The authors declare that all the relevant data, associated protocols, and materials supporting the findings of this study are present in the paper.

## References

[1] Evans RM , Mangelsdorf DJ . Nuclear receptors, RXR, and the big bang. Cell. 2014;157:255‐266. 24679540 10.1016/j.cell.2014.03.012 PMC4029515

[2] Sharma S , Shen T , Chitranshi N , et al. Retinoid X receptor: cellular and biochemical roles of nuclear receptor with a focus on neuropathological involvement. Mol Neurobiol. 2022;59:2027‐2050. 35015251 10.1007/s12035-021-02709-y PMC9015987

[3] Gilardi F , Desvergne B . RXRs: Collegial Partners. In: Asson‐Batres MA , Rochette‐Egly C , eds. The Biochemistry of Retinoic Acid Receptors I: Structure, Activation, and Function at the Molecular Level. Springer Netherlands; 2014:75‐102. 10.1007/978-94-017-9050-5_5 24962882

[4] Osz J , McEwen AG , Poussin‐Courmontagne P , et al. Structural basis of natural promoter recognition by the retinoid X nuclear receptor. Sci Rep. 2015;5:8216. 25645674 10.1038/srep08216 PMC4314640

[5] Rastinejad F . Retinoic acid receptor structures: the journey from single domains to full‐length complex. J Mol Endocrinol. 2022;69:T25‐T36. 36069789 10.1530/JME-22-0113 PMC11376212

[6] Krężel W , Rühl R , de Lera AR . Alternative retinoid X receptor (RXR) ligands. Mol Cell Endocrinol. 2019;491:110436. 31026478 10.1016/j.mce.2019.04.016

[7] Dawson MI , Xia Z . The retinoid X receptors and their ligands. Biochim Biophys Acta. 2012;1821:21‐56. 22020178 10.1016/j.bbalip.2011.09.014 PMC4097889

[8] Heyman RA , Mangelsdorf DJ , Dyck JA , et al. 9‐cis retinoic acid is a high affinity ligand for the retinoid X receptor. Cell. 1992;68:397‐406. 1310260 10.1016/0092-8674(92)90479-v

[9] Allenby G , Bocquel MT , Saunders M , et al. Retinoic acid receptors and retinoid X receptors: interactions with endogenous retinoic acids. Proc Natl Acad Sci USA. 1993;90(1):30‐34. doi:10.1073/pnas.90.1.30 8380496 PMC45593

[10] Ruhl R , Krzyzosiak A , Niewiadomska‐Cimicka A , et al. 9‐cis‐13,14‐Dihydroretinoic acid is an endogenous retinoid acting as RXR ligand in mice. PLoS Genet. 2015;11:e1005213. 26030625 10.1371/journal.pgen.1005213 PMC4451509

[11] Krzyzosiak A , Podlesny‐Drabiniok A , Vaz B , et al. Vitamin A5/X controls stress‐adaptation and prevents depressive‐like behaviors in a mouse model of chronic stress. Neurobiol Stress. 2021;15:100375. 34401411 10.1016/j.ynstr.2021.100375 PMC8355947

[12] Krezel W , Rivas A , Szklenar M , et al. Vitamin A5/X, a new food to lipid hormone concept for a nutritional ligand to control RXR‐mediated signaling. Nutrients. 2021;13(3):925. 33809241 10.3390/nu13030925 PMC7999121

[13] de Lera AR , Krezel W , Ruhl R . An endogenous mammalian retinoid X receptor ligand, at last! ChemMedChem. 2016;11:1027‐1037. 27151148 10.1002/cmdc.201600105

[14] Rühl R , Krezel W , de Lera AR . 9‐Cis‐13,14‐dihydroretinoic acid, a new endogenous mammalian ligand of retinoid X receptor and the active ligand of a potential new vitamin a category: vitamin A5. Nutr Rev. 2018;76:929‐941. 30358857 10.1093/nutrit/nuy057

[15] Das BC , Dasgupta S , Ray SK . Potential therapeutic roles of retinoids for prevention of neuroinflammation and neurodegeneration in Alzheimer's disease. Neural Regen Res. 2019;14:1880‐1892. 31290437 10.4103/1673-5374.259604 PMC6676868

[16] Clark JN , Whiting A , McCaffery P . Retinoic acid receptor‐targeted drugs in neurodegenerative disease. Expert Opin Drug Metab Toxicol. 2020;16:1097‐1108. 32799572 10.1080/17425255.2020.1811232

[17] Wood H . Retinoid X receptor mediates brain clean‐up after stroke. Nat Rev Neurol. 2020;16:128‐129. 10.1038/s41582-020-0315-9 31988486

[18] Willems S , Zaienne D , Merk D . Targeting nuclear receptors in neurodegeneration and Neuroinflammation. J Med Chem. 2021;64:9592‐9638. 34251209 10.1021/acs.jmedchem.1c00186

[19] Dheer Y , Chitranshi N , Gupta V , et al. Retinoid x receptor modulation protects against ER stress response and rescues glaucoma phenotypes in adult mice. Exp Neurol. 2019;314:111‐125. 30703361 10.1016/j.expneurol.2019.01.015

[20] Huang JK , Jarjour AA , Nait Oumesmar B , et al. Retinoid X receptor gamma signaling accelerates CNS remyelination. Nat Neurosci. 2011;14:45‐53. 21131950 10.1038/nn.2702 PMC4013508

[21] Jayaram H , Kolko M , Friedman DS , Gazzard G . Glaucoma: now and beyond. Lancet. 2023;402:1788‐1801. 37742700 10.1016/S0140-6736(23)01289-8

[22] You Y , Gupta VK , Li JC , Klistorner A , Graham SL . Optic neuropathies: characteristic features and mechanisms of retinal ganglion cell loss. Rev Neurosci. 2013;24:301‐321. 23612594 10.1515/revneuro-2013-0003

[23] Shen J , Wang Y , Yao K . Protection of retinal ganglion cells in glaucoma: current status and future. Exp Eye Res. 2021;205:108506. 33609512 10.1016/j.exer.2021.108506

[24] Basavarajappa D , Galindo‐Romero C , Gupta V , et al. Signalling pathways and cell death mechanisms in glaucoma: insights into the molecular pathophysiology. Mol Asp Med. 2023;94:101216. 10.1016/j.mam.2023.101216 37856930

[25] Tezel G . A broad perspective on the molecular regulation of retinal ganglion cell degeneration in glaucoma. Prog Brain Res. 2020;256:49‐77. 32958215 10.1016/bs.pbr.2020.05.027 PMC11822681

[26] Syc‐Mazurek SB , Libby RT . Axon injury signaling and compartmentalized injury response in glaucoma. Prog Retin Eye Res. 2019;73:100769. 31301400 10.1016/j.preteyeres.2019.07.002 PMC6898776

[27] Volonté YA , Ayala‐Peña VB , Vallese‐Maurizi H , et al. Retinoid X receptor activation promotes photoreceptor survival and modulates the inflammatory response in a mouse model of retinitis pigmentosa. Biochim Biophys Acta, Mol Cell Res. 2021;1868:119098. 34271041 10.1016/j.bbamcr.2021.119098

[28] Kastner P , Mark M , Ghyselinck N , et al. Genetic evidence that the retinoid signal is transduced by heterodimeric RXR/RAR functional units during mouse development. Development. 1997;124:313‐326. 9053308 10.1242/dev.124.2.313

[29] Dheer Y , Chitranshi N , Gupta V , et al. Bexarotene modulates retinoid‐X‐receptor expression and is protective against neurotoxic endoplasmic reticulum stress response and apoptotic pathway activation. Mol Neurobiol. 2018;55:9043‐9056. 29637440 10.1007/s12035-018-1041-9

[30] Basavarajappa D , Gupta V , Chitranshi N , et al. Anti‐inflammatory effects of Siponimod in a mouse model of Excitotoxicity‐induced retinal injury. Mol Neurobiol. 2023;60:7222‐7237. 37542647 10.1007/s12035-023-03535-0 PMC10657799

[31] Chitranshi N , Rajput R , Godinez A , et al. Neuroserpin gene therapy inhibits retinal ganglion cell apoptosis and promotes functional preservation in glaucoma. Mol Ther. 2023;31:2056‐2076. 36905120 10.1016/j.ymthe.2023.03.008 PMC10362384

[32] Basavarajappa D , Gupta V , Chitranshi N , et al. Siponimod exerts neuroprotective effects on the retina and higher visual pathway through neuronal S1PR1 in experimental glaucoma. Neural Regen Res. 2023;18:840‐848. 36204852 10.4103/1673-5374.344952 PMC9700103

[33] Basavarajappa D , Gupta V , Wall RV , et al. S1PR1 signaling attenuates apoptosis of retinal ganglion cells via modulation of cJun/Bim cascade and bad phosphorylation in a mouse model of glaucoma. FASEB J. 2023;37:e22710. 36520045 10.1096/fj.202201346R PMC13281836

[34] Chitranshi N , Dheer Y , Mirzaei M , et al. Loss of Shp2 rescues BDNF/TrkB signaling and contributes to improved retinal ganglion cell neuroprotection. Mol Ther. 2019;27:424‐441. 30341011 10.1016/j.ymthe.2018.09.019 PMC6369445

[35] Thananthirige KPM , Chitranshi N , Basavarajappa D , et al. Tau modulation through AAV9 therapy augments Akt/Erk survival signalling in glaucoma mitigating the retinal degenerative phenotype. Acta Neuropathol Commun. 2024;12:89. 38845058 10.1186/s40478-024-01804-0 PMC11158005

[36] Saszik SM , Robson JG , Frishman LJ . The scotopic threshold response of the dark‐adapted electroretinogram of the mouse. J Physiol. 2002;543:899‐916. 12231647 10.1113/jphysiol.2002.019703 PMC2290546

[37] Gupta VK , Gowda LR . Alpha‐1‐proteinase inhibitor is a heparin binding serpin: molecular interactions with the Lys rich cluster of helix‐F domain. Biochimie. 2008;90:749‐761. 18261994 10.1016/j.biochi.2008.01.004

[38] Basavarajappa DK , Gupta VK , Dighe R , Rajala A , Rajala RV . Phosphorylated Grb14 is an endogenous inhibitor of retinal protein tyrosine phosphatase 1B, and light‐dependent activation of Src phosphorylates Grb14. Mol Cell Biol. 2011;31:3975‐3987. 21791607 10.1128/MCB.05659-11 PMC3187357

[39] Gupta VK , Rajala A , Rajala RV . Insulin receptor regulates photoreceptor CNG channel activity. Am J Physiol Endocrinol Metab. 2012;303:E1363‐E1372. 23032687 10.1152/ajpendo.00199.2012 PMC3774084

[40] Abbasi M , Gupta VK , Chitranshi N , et al. Caveolin‐1 ablation imparts partial protection against inner retinal injury in experimental glaucoma and reduces apoptotic activation. Mol Neurobiol. 2020;57:3759‐3784. 32578008 10.1007/s12035-020-01948-9

[41] Chidlow G , Ebneter A , Wood JP , Casson RJ . The optic nerve head is the site of axonal transport disruption, axonal cytoskeleton damage and putative axonal regeneration failure in a rat model of glaucoma. Acta Neuropathol. 2011;121:737‐751. 21311901 10.1007/s00401-011-0807-1 PMC3098991

[42] Kroeger H , Chiang WC , Felden J , Nguyen A , Lin JH . ER stress and unfolded protein response in ocular health and disease. FEBS J. 2019;286:399‐412. 29802807 10.1111/febs.14522 PMC6583901

[43] Wang LH , Chen GL , Chen K , et al. Dual targeting of retinoid X receptor and histone deacetylase with DW22 as a novel antitumor approach. Oncotarget. 2015;6:9740‐9755. 25762635 10.18632/oncotarget.3149 PMC4496394

[44] Wang W , Zhao M , Cui L , et al. Characterization of a novel HDAC/RXR/HtrA1 signaling axis as a novel target to overcome cisplatin resistance in human non‐small cell lung cancer. Mol Cancer. 2020;19:134. 32878625 10.1186/s12943-020-01256-9 PMC7466461

[45] Lebrun‐Julien F , Suter U . Combined HDAC1 and HDAC2 depletion promotes retinal ganglion cell survival after injury through reduction of p53 target gene expression. ASN Neuro. 2015;7(3):1759091415593066. 26129908 10.1177/1759091415593066 PMC4720215

[46] Crosson CE , Mani SK , Husain S , Alsarraf O , Menick DR . Inhibition of histone deacetylase protects the retina from ischemic injury. Invest Ophthalmol Vis Sci. 2010;51:3639‐3645. 20164449 10.1167/iovs.09-4538 PMC2904015

[47] Dummer R , Beyer M , Hymes K , et al. Vorinostat combined with bexarotene for treatment of cutaneous T‐cell lymphoma: in vitro and phase I clinical evidence supporting augmentation of retinoic acid receptor/retinoid X receptor activation by histone deacetylase inhibition. Leuk Lymphoma. 2012;53:1501‐1508. 22239668 10.3109/10428194.2012.656625

[48] Alsarraf O , Fan J , Dahrouj M , Chou CJ , Yates PW , Crosson CE . Acetylation preserves retinal ganglion cell structure and function in a chronic model of ocular hypertension. Invest Ophthalmol Vis Sci. 2014;55:7486‐7493. 25358731 10.1167/iovs.14-14792 PMC4240722

[49] Levkovitch‐Verbin H . Retinal ganglion cell apoptotic pathway in glaucoma: initiating and downstream mechanisms. Prog Brain Res. 2015;220:37‐57. 26497784 10.1016/bs.pbr.2015.05.005

[50] Hollville E , Romero SE , Deshmukh M . Apoptotic cell death regulation in neurons. FEBS J. 2019;286:3276‐3298. 31230407 10.1111/febs.14970 PMC6718311

[51] Maes ME , Schlamp CL , Nickells RW . BAX to basics: how the BCL2 gene family controls the death of retinal ganglion cells. Prog Retin Eye Res. 2017;57:1‐25. 28064040 10.1016/j.preteyeres.2017.01.002 PMC5350025

[52] Wang N , Tall AR . Regulation and mechanisms of ATP‐binding cassette transporter A1‐mediated cellular cholesterol efflux. Arterioscler Thromb Vasc Biol. 2003;23:1178‐1184. 12738681 10.1161/01.ATV.0000075912.83860.26

[53] Murthy S , Born E , Mathur SN , Field FJ . LXR/RXR activation enhances basolateral efflux of cholesterol in CaCo‐2 cells. J Lipid Res. 2002;43:1054‐1064. 12091489 10.1194/jlr.m100358-jlr200

[54] Koldamova RP , Lefterov IM , Ikonomovic MD , et al. 22R‐hydroxycholesterol and 9‐cis‐retinoic acid induce ATP‐binding cassette transporter A1 expression and cholesterol efflux in brain cells and decrease amyloid β secretion*. J Biol Chem. 2003;278:13244‐13256. 12547833 10.1074/jbc.M300044200

[55] Yang J , Chen Y , Zou T , et al. Cholesterol homeostasis regulated by ABCA1 is critical for retinal ganglion cell survival. Sci China Life Sci. 2023;66:211‐225. 35829808 10.1007/s11427-021-2126-2

[56] Chen Y , Lin Y , Vithana EN , et al. Common variants near ABCA1 and in PMM2 are associated with primary open‐angle glaucoma. Nat Genet. 2014;46:1115‐1119. 25173107 10.1038/ng.3078

[57] Baudouin C , Kolko M , Melik‐Parsadaniantz S , Messmer EM . Inflammation in glaucoma: from the back to the front of the eye, and beyond. Prog Retin Eye Res. 2021;83:100916. 33075485 10.1016/j.preteyeres.2020.100916

[58] Mélik Parsadaniantz S , le Réaux‐Goazigo A , Sapienza A , Habas C , Baudouin C . Glaucoma: a degenerative optic neuropathy related to Neuroinflammation? Cells. 2020;9(3):535. doi:10.3390/cells9030535 32106630 PMC7140467

[59] Mori M , Ghyselinck NB , Chambon P , Mark M . Systematic immunolocalization of retinoid receptors in developing and adult mouse eyes. Invest Ophthalmol Vis Sci. 2001;42:1312‐1318. 11328745

[60] Gorbatyuk MS , Starr CR , Gorbatyuk OS . Endoplasmic reticulum stress: new insights into the pathogenesis and treatment of retinal degenerative diseases. Prog Retin Eye Res. 2020;79:100860. 32272207 10.1016/j.preteyeres.2020.100860 PMC7541398

[61] Anholt RR , Carbone MA . A molecular mechanism for glaucoma: endoplasmic reticulum stress and the unfolded protein response. Trends Mol Med. 2013;19:586‐593. 23876925 10.1016/j.molmed.2013.06.005 PMC3795998

[62] Zode GS , Sharma AB , Lin X , et al. Ocular‐specific ER stress reduction rescues glaucoma in murine glucocorticoid‐induced glaucoma. J Clin Invest. 2014;124:1956‐1965. 24691439 10.1172/JCI69774 PMC4001532

[63] Riancho J , Ruiz‐Soto M , Berciano MT , Berciano J , Lafarga M . Neuroprotective effect of Bexarotene in the SOD1(G93A) mouse model of amyotrophic lateral sclerosis. Front Cell Neurosci. 2015;9:250. 26190974 10.3389/fncel.2015.00250 PMC4486838

[64] Li Y , Shen Q , Kim HT , et al. The rexinoid bexarotene represses cyclin D1 transcription by inducing the DEC2 transcriptional repressor. Breast Cancer Res Treat. 2011;128:667‐677. 20821348 10.1007/s10549-010-1083-9 PMC3444826

[65] Schmitt HM , Schlamp CL , Nickells RW . Role of HDACs in optic nerve damage‐induced nuclear atrophy of retinal ganglion cells. Neurosci Lett. 2016;625:11‐15. 26733303 10.1016/j.neulet.2015.12.012 PMC5125391

[66] Schlüter A , Aksan B , Fioravanti R , Valente S , Mai A , Mauceri D . Histone deacetylases contribute to Excitotoxicity‐triggered degeneration of retinal ganglion cells in vivo. Mol Neurobiol. 2019;56:8018‐8034. 31161423 10.1007/s12035-019-01658-x

[67] Schmitt HM , Schlamp CL , Nickells RW . Targeting HDAC3 activity with RGFP966 protects against retinal ganglion cell nuclear atrophy and apoptosis after optic nerve injury. J Ocul Pharmacol Ther. 2018;34:260‐273. 29211617 10.1089/jop.2017.0059 PMC5963665

[68] Zaidi SAH , Guzman W , Singh S , Mehrotra S , Husain S . Changes in class I and IIb HDACs by delta‐opioid in chronic rat glaucoma model. Invest Ophthalmol Vis Sci. 2020;61:4. 10.1167/iovs.61.14.4 PMC7718808 33263714

[69] Paseban T , Alavi MS , Etemad L , Roohbakhsh A . The role of the ATP‐binding cassette A1 (ABCA1) in neurological disorders: a mechanistic review. Expert Opin Ther Targets. 2023;27:531‐552. 37428709 10.1080/14728222.2023.2235718

[70] Lewandowski CT , Laham MS , Thatcher GRJ . Remembering your a, B, C's: Alzheimer's disease and ABCA1. Acta Pharm Sin B. 2022;12:995‐1018. 35530134 10.1016/j.apsb.2022.01.011 PMC9072248

[71] Jacobo‐Albavera L , Domínguez‐Pérez M , Medina‐Leyte DJ , González‐Garrido A , Villarreal‐Molina T . The role of the ATP‐binding cassette A1 (ABCA1) in human disease. Int J Mol Sci. 2021;22(4):1593. 33562440 10.3390/ijms22041593 PMC7915494

[72] Kiss M , Czimmerer Z , Nagy G , et al. Retinoid X receptor suppresses a metastasis‐promoting transcriptional program in myeloid cells via a ligand‐insensitive mechanism. Proc Natl Acad Sci USA. 2017;114:10725‐10730. 28923935 10.1073/pnas.1700785114 PMC5635866

[73] Mukwaya A , Lennikov A , Xeroudaki M , et al. Time‐dependent LXR/RXR pathway modulation characterizes capillary remodeling in inflammatory corneal neovascularization. Angiogenesis. 2018;21:395‐413. 29445990 10.1007/s10456-018-9604-y PMC5878196

[74] Natrajan MS , de la Fuente AG , Crawford AH , et al. Retinoid X receptor activation reverses age‐related deficiencies in myelin debris phagocytosis and remyelination. Brain. 2015;138:3581‐3597. 26463675 10.1093/brain/awv289 PMC4668920

[75] Huang JK , Franklin RJ . Regenerative medicine in multiple sclerosis: identifying pharmacological targets of adult neural stem cell differentiation. Neurochem Int. 2011;59:329‐332. 21300122 10.1016/j.neuint.2011.01.017

[76] Zuo Y , Huang L , Enkhjargal B , et al. Activation of retinoid X receptor by bexarotene attenuates neuroinflammation via PPARγ/SIRT6/FoxO3a pathway after subarachnoid hemorrhage in rats. J Neuroinflammation. 2019;16:47. 30791908 10.1186/s12974-019-1432-5 PMC6385420

[77] Kirchmeyer M , Koufany M , Sebillaud S , Netter P , Jouzeau JY , Bianchi A . All‐trans retinoic acid suppresses interleukin‐6 expression in interleukin‐1‐stimulated synovial fibroblasts by inhibition of ERK1/2 pathway independently of RAR activation. Arthritis Res Ther. 2008;10:R141. 19068145 10.1186/ar2569 PMC2656246

[78] Li Y , Xing Q , Wei Y , et al. Activation of RXR by bexarotene inhibits inflammatory conditions in human rheumatoid arthritis fibroblast‐like synoviocytes. Int J Mol Med. 2019;44:1963‐1970. 31545398 10.3892/ijmm.2019.4336

[79] le Maire A , Germain P , Bourguet W . Protein‐protein interactions in the regulation of RAR–RXR heterodimers transcriptional activity. In: Pohl E , ed. Methods in Enzymology. Vol 637. Academic Press; 2020:175‐207. 32359645 10.1016/bs.mie.2020.02.007

[80] Botling J , Castro DS , Öberg F , Nilsson K , Perlmann T . Retinoic acid receptor/retinoid X receptor heterodimers can be activated through both subunits providing a basis for synergistic transactivation and cellular differentiation. J Biol Chem. 1997;272:9443‐9449. 9083083 10.1074/jbc.272.14.9443

[81] Samarut E , Rochette‐Egly C . Nuclear retinoic acid receptors: conductors of the retinoic acid symphony during development. Mol Cell Endocrinol. 2012;348:348‐360. 21504779 10.1016/j.mce.2011.03.025

