# Supplementary material for: Retinoid X receptor agonist 9CDHRA mitigates retinal ganglion cell apoptosis and neuroinflammation in a mouse model of glaucoma
Source: FASEB J. 2025 Mar 13;39(6):e70465. doi: 10.1096/fj.202402642R (PMC11904862; doi:10.1096/fj.202402642R)

Supplementary file for

Retinoid X Receptor Agonist 9CDHRA Mitigates Retinal Ganglion Cell Apoptosis and Neuroinflammation in a Mouse Model of Glaucoma

This file includes:

Supplemental **Figures S1-S3**

Supplementary Figure S1.

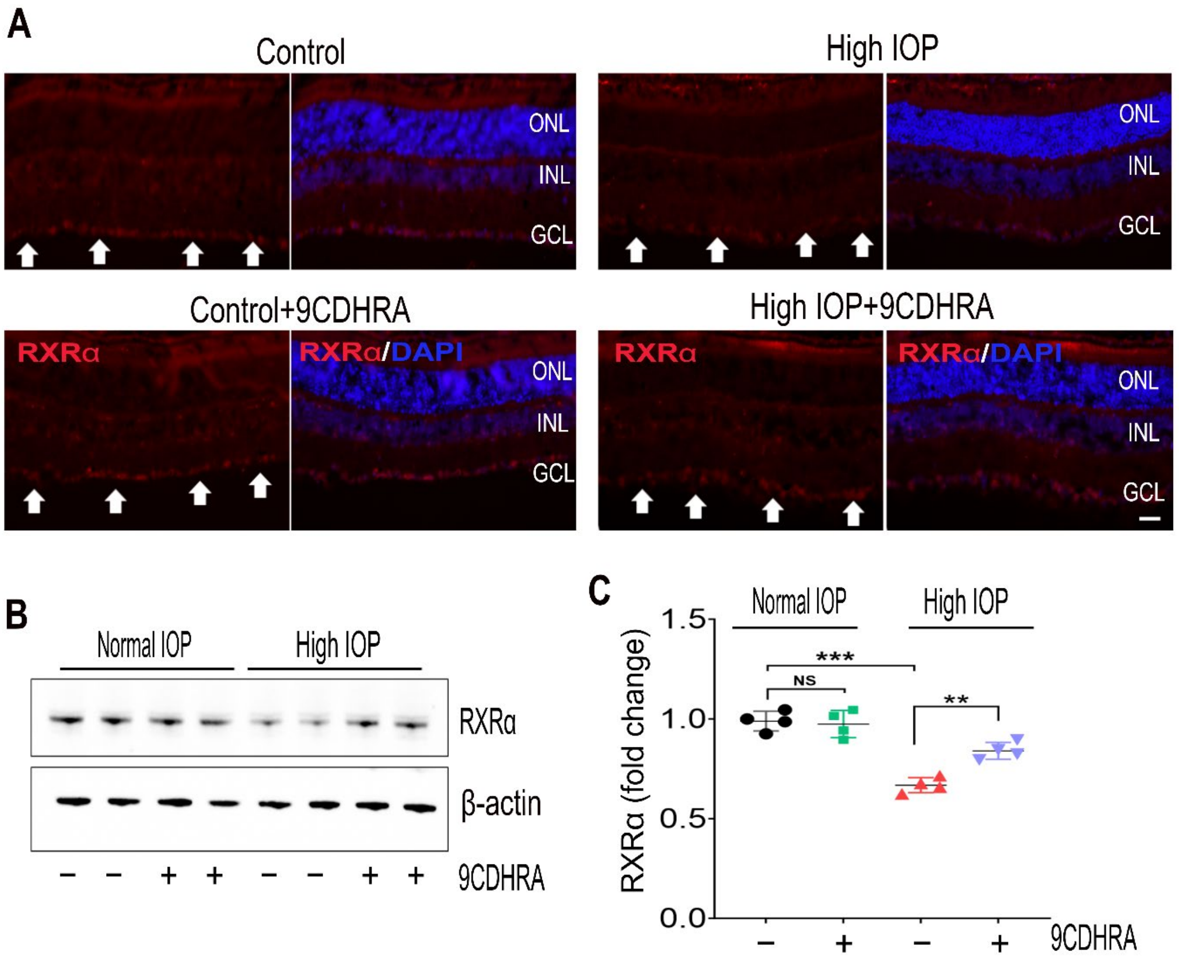

Figure S1. Analysis of RXRα expression changes in the retinal tissues. (A) Immunofluorescence images of retinal sections stained with RXRα (red) and DAPI (blue) (representative images, scale bar: 50 μm; ONL, outer nuclear layer; INL, inner nuclear layer; GCL, ganglion cell layer) demonstrate changes in RXRα expression and its modulation with 9CDHRA treatment in high-IOP retinas (arrows indicate changes in RXRα immunoreactivity). (B) Western blot analysis of retinal tissues for RXRα levels following high-IOP injury and 9CDHRA treatment (representative blots). (C) Densitometric quantitative analysis of RXRα blot densities (normalized to β-actin) reveals decreased RXRα levels in high-IOP retinas and a significant protection with 9CDHRA treatment (NS, not significant, $**P < 0.01$, $***P < 0.001$, one-way ANOVA with Tukey's multiple comparisons test, n = 4 per group).

Supplementary Figure S2. Specificity of antibody immunoreactivity validated through experimental controls.

Immunofluorescence images of optic nerve sections stained with pNFH.

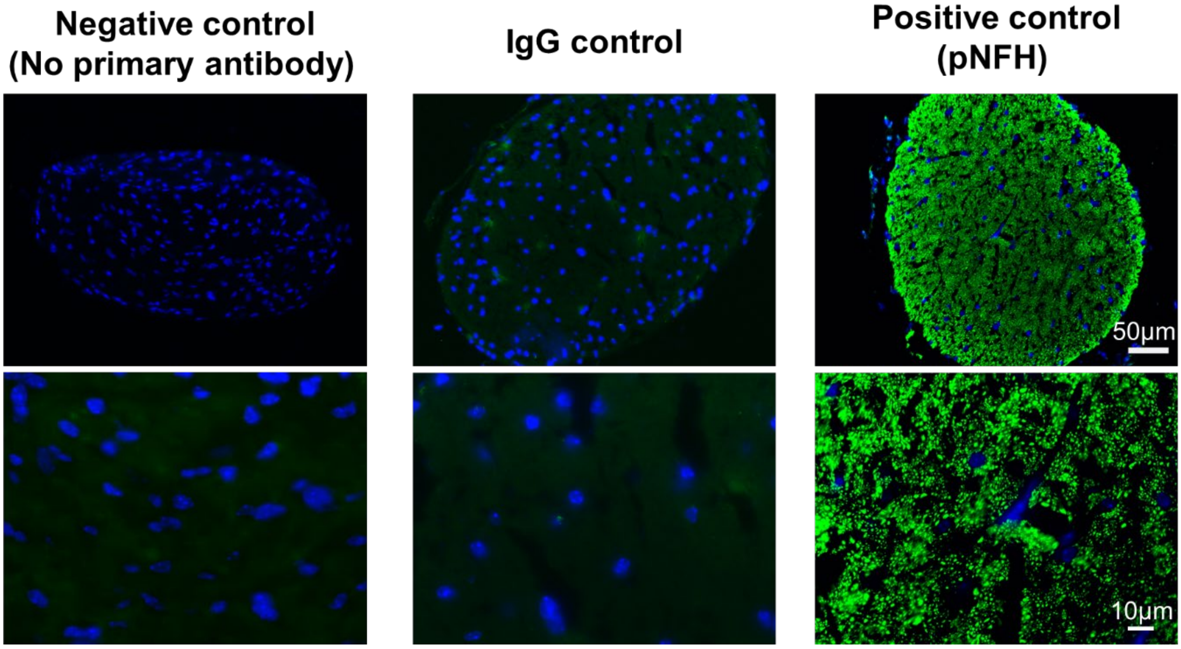

Supplementary Figure S2 continued.

Immunofluorescence images of optic nerve sections stained with Iba1

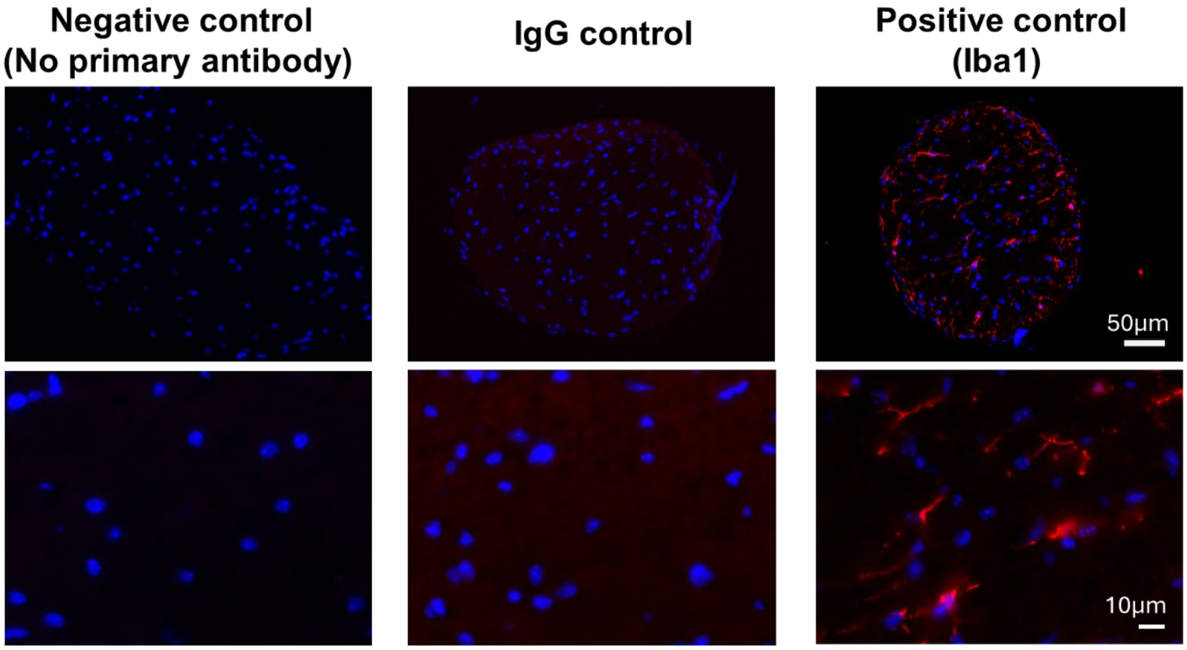

Supplementary Figure S2 continued.

Immunofluorescence images of optic nerve sections stained with GFAP.

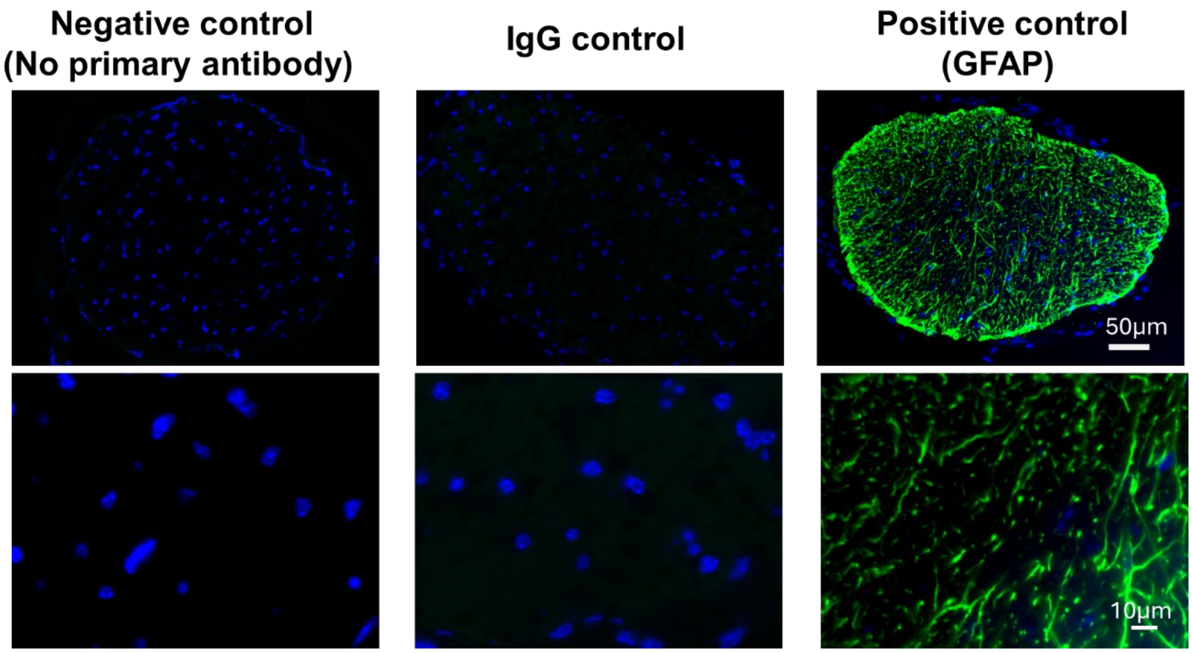

Supplementary Figure S3. Raw data of Western blots.

Raw blot data related to Figure 3

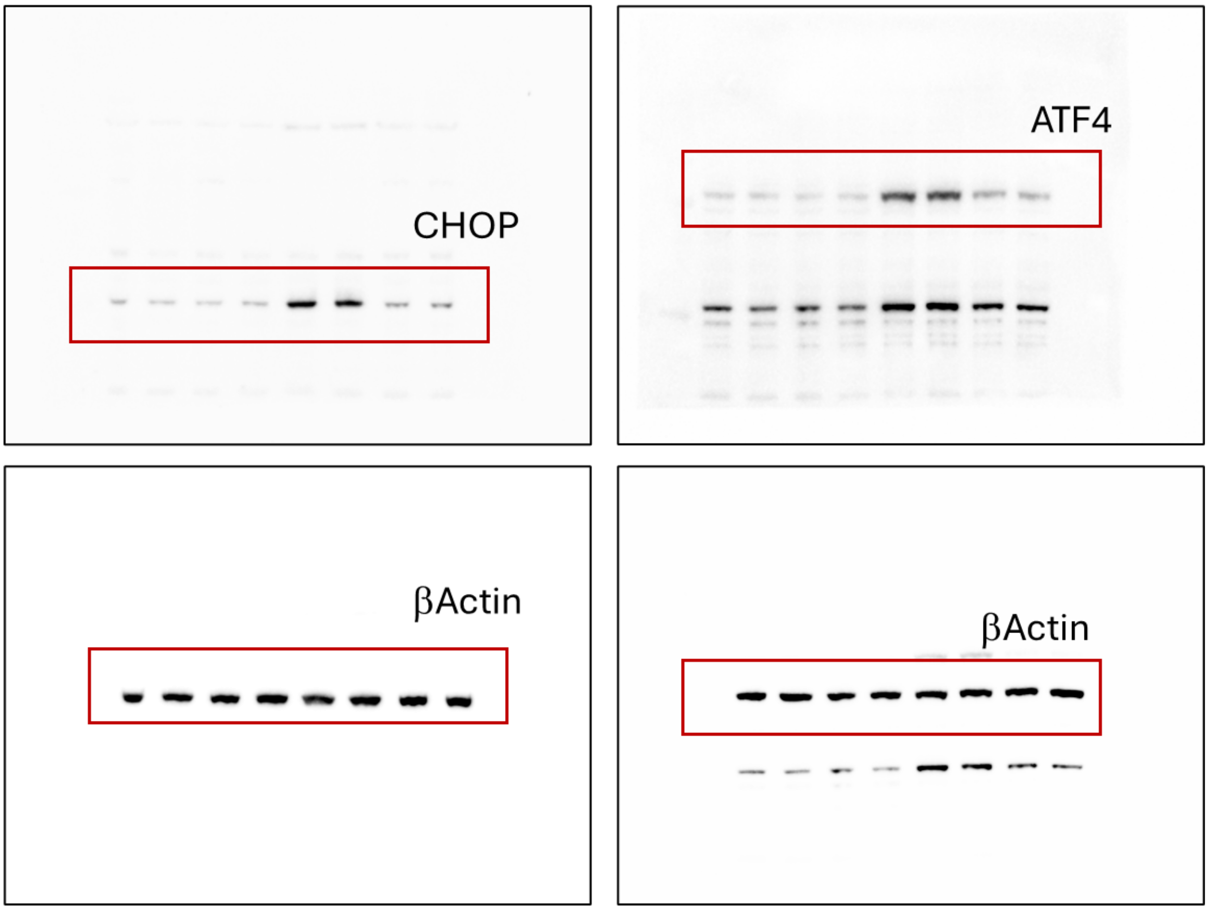

Supplementary Figure S3 continued.

Raw blot data related to Figure 5

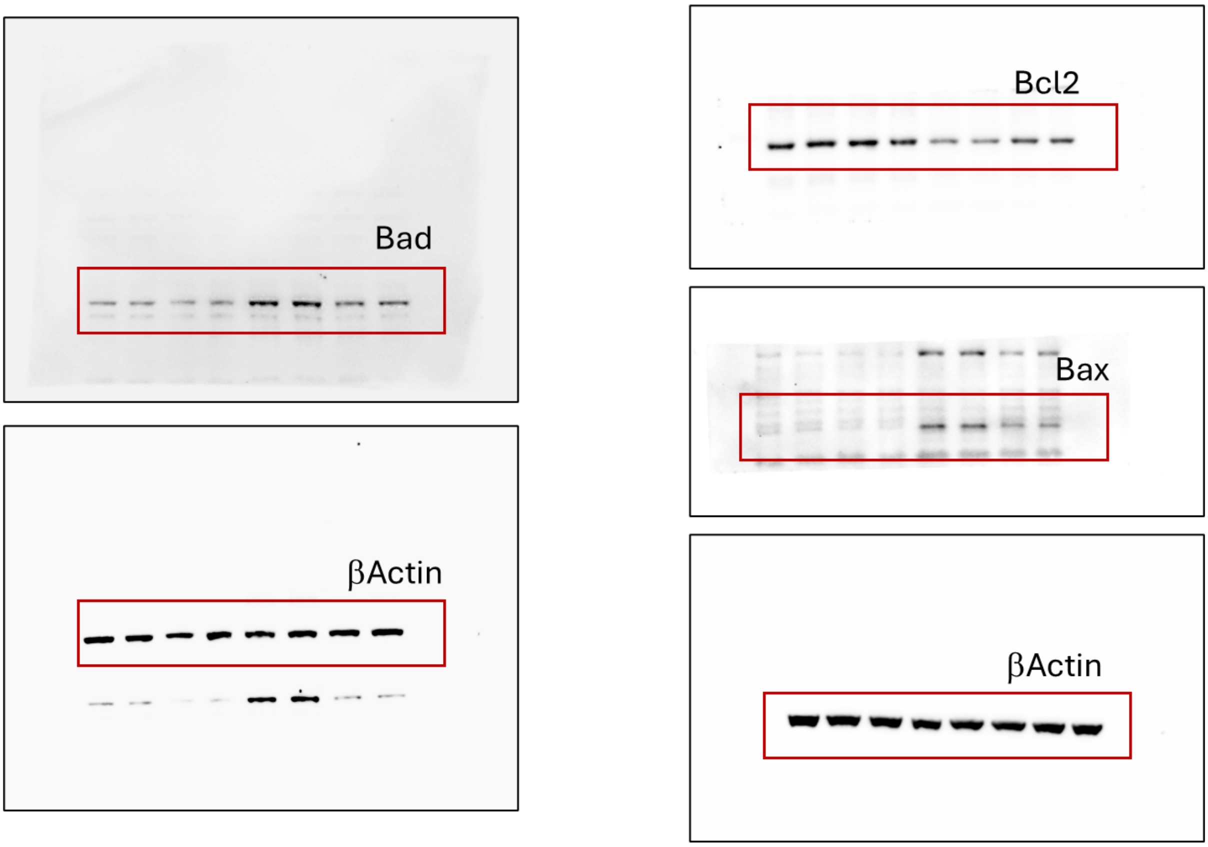

Supplementary Figure 3 continued.

Raw blot data related to Figure 6

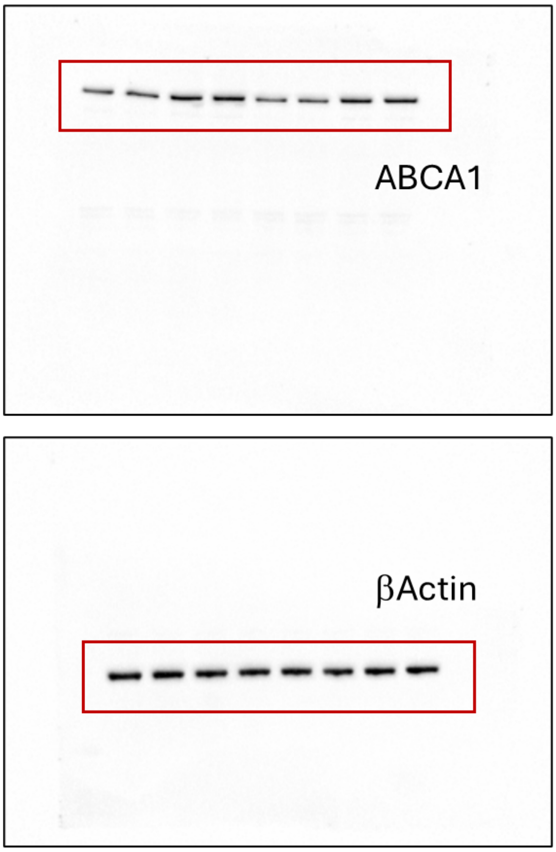

Supplementary Figure 3 continued.

Raw blot data related to Figure 7

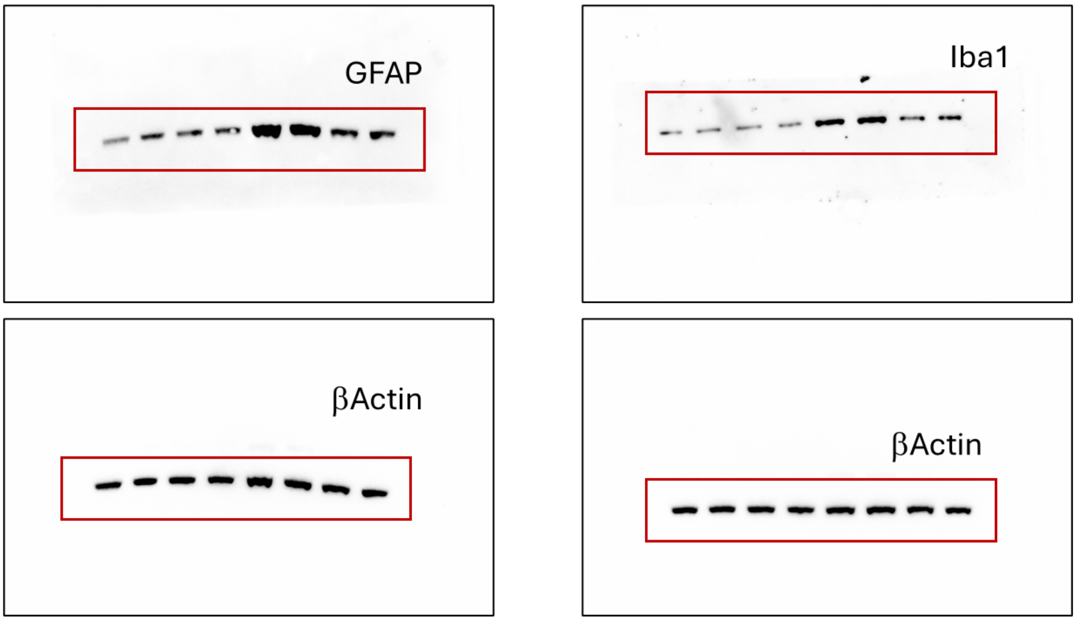

Supplementary Figure 3 continued.

Raw blot data related to Figure S1

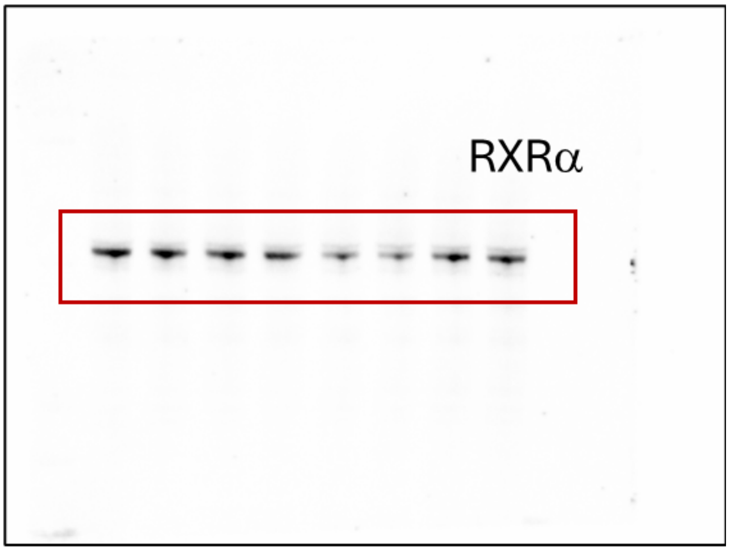

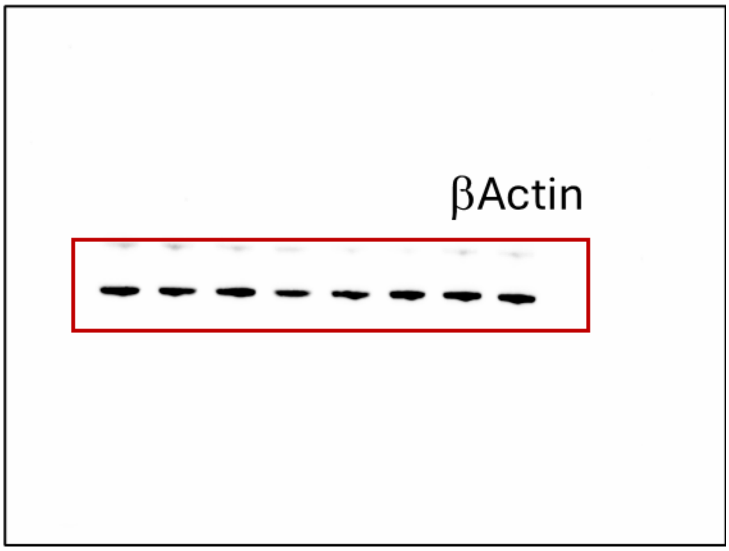

Supplement: Supplementary file 1 — Figure S1. [file FSB2-39-e70465-s001.pdf]
